# Engineered extracellular vesicles for combinatorial cancer therapy and imaging

**DOI:** 10.1016/j.apsb.2026.03.054

**Published:** 2026-04-03

**Authors:** Anita S. La'ah, Nashmin Fayazi Hosseini, Choongmo Kang, Amirhossein Ahmadieh-Yazdi, Mohsen Sheykhhasan, Piao Yang, Armita Ghotaslou, Hamed Manoochehri, Yun Suk Huh, Heemin Kang, Pooyan Makvandi

**Affiliations:** a Taiwan International Graduate Program in Molecular Medicine, National Yang Ming Chiao Tung University and Academia Sinica, Taipei 115, China; b Research Center for Molecular Medicine, Hamadan University of Medical Sciences, Hamadan 6517838736, Iran; c Department of Materials Science and Engineering, Korea University, Seoul 02841, Republic of Korea; d Stem Cell Biology Research Center, Yazd Reproductive Sciences Institute, Shahid Sadoughi University of Medical Sciences, Yazd 8916978477, Iran; e Cellular and Molecular Research Center, Qom University of Medical Sciences, Qom, Iran; f Department of Molecular Genetics, College of Arts and Sciences, The Ohio State University, Columbus, OH 43210, USA; g Department of Medical Biotechnology, Faculty of Advanced Medical Sciences, Tabriz University of Medical Sciences, Tabriz 51385-1444, Iran; h The Persian Gulf Marine Biotechnology Research Center, The Persian Gulf Biomedical Sciences Research Institute, Bushehr University of Medical Sciences, Bushehr 7514633196, Iran; i NanoBio High-Tech Materials Research Center, Department of Biological Sciences and Bioengineering, Inha University, Incheon 22212, Republic of Korea; j College of Medicine, Korea University, Seoul 02841, Republic of Korea; k The Quzhou Affiliated Hospital of Wenzhou Medical University, Quzhou People’s Hospital, 324000 Quzhou, China; l Centre for Research Impact and Outcome, Chitkara University, Rajpura 140401, Punjab, India; m University College, Korea University, Seoul 02841, Republic of Korea

**Keywords:** Modified extracellular vesicles, Cancer diagnosis, Cancer therapy, Imaging-guided therapy, Gene therapy, Immunotherapy, Drug delivery, Combination therapy

## Abstract

Cancer treatment has advanced significantly over the past few decades, resulting in improved patient survival outcomes. However, challenges like drug resistance, off-target effects, and systemic toxicity continue to persist. These underscore the importance of modified extracellular vesicles (EVs) as a versatile and innovative platform for delivering combination therapies for cancer. This review highlights the utilization of biocompatible engineered EVs to inhibit cancer progression with reduced side effects. Further, outlining the promising approach to cancer treatment through combination therapies and imaging-guided strategies. Additionally, this review explored the biogenesis and various sources of EVs, which provides clear insights into future directions.

## Graphical abstract

Extracellular vesicles (EVs), through various engineered modifications, serve as a versatile and innovative platform enabling combination cancer therapies and diagnostic applications.

**Figure:**
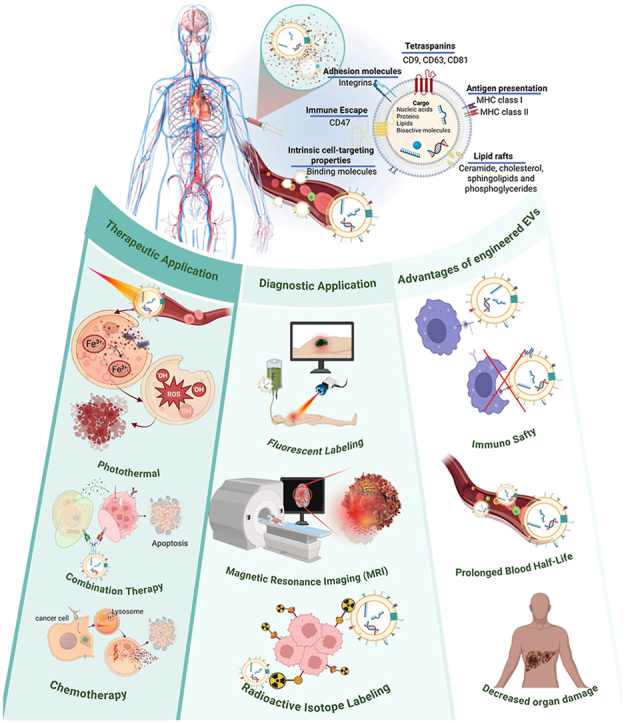

## 1 Introduction

Despite major advancements in conventional cancer treatments like surgery, radiotherapy, chemotherapy, and hormonal therapy, their long-term effectiveness remains limited due to drug resistance, systemic toxicity, incomplete tumor removal, and recurrence. Hence, advanced techniques such as fluorescence-guided surgery and image-guided radiotherapy have enhanced treatment precision^1^. The groundbreaking discoveries of Johnstone and Stahl, who identified exosomes as a mechanism for shedding obsolete proteins, marked the beginning of the foundational period in the early 1980s^2^. They described exosomes as vesicles released by developing reticulocytes^2^. This work delineated the composition of extracellular vesicles (EVs) and the measurement of their prevalence in diseases throughout the 1980s and 1990s^2^. In 1996, researchers discovered that immune cell EVs could deliver antigens, inspiring interest in their potential for novel delivery systems and regenerative therapies as alternatives to traditional cell-based treatments^2^.

EVs are membrane-bound vesicles released by cells^3^^,^^4^. They are employed in various functions, such as nanovaccines, immune reprogramming, checkpoint blockade and inducing cytotoxicity^5^^,^^6^. Compared to conventional nanocarriers, EVs offer better biocompatibility, extended circulation time, and lower immunogenicity and toxicity^4^^,^^7^^,^^8^. EV engineering can be achieved through modifying donor cells before EV isolation or directly altering the EVs after they have been isolated^9^, ^10^, ^11^, ^12^. Direct modification of EVs involves techniques like chemical coupling, noncovalent attachment, membrane fusion, or extrusion to incorporate new molecules or nanomaterials into EV^13^, ^14^, ^15^. In contrast, donor cell modification involves genetic engineering, chemical treatment, or physical stimuli to alter the content size, surface, or shape of the EVs^16^, ^17^, ^18^.

In combinational cancer therapy, engineered EVs are suitable for multiple cancer types. Combination of engineered EVs with chemotherapy enhances treatment efficacy and minimizes side effects by co-delivering drugs with agents like photosensitizers or gene regulators^4^^,^^16^^,^^19^, ^20^, ^21^. EV-immunotherapy combinations act as a nanovaccines by presenting tumor-associated antigens to stimulate adaptive immunity^5^^,^^22^^,^^23^. They can also carry immune modulators like cytokines, checkpoint inhibitors, or small interfering RNAs (siRNAs) to reprogram the tumor microenvironment (TME) and counteract immunosuppression^4^^,^^16^^,^^24^^,^^25^. Additionally, EVs can be loaded with visualizing agents, including quantum dots (QDs), magnetic nanoparticles (NPs), or fluorescent dyes to visualize tumor tissues and monitor imaging-guided therapy^4^^,^^16^^,^^26^.

## 2 Extracellular vesicles

### 2.1 EV biogenesis and release

**Figure 1:**
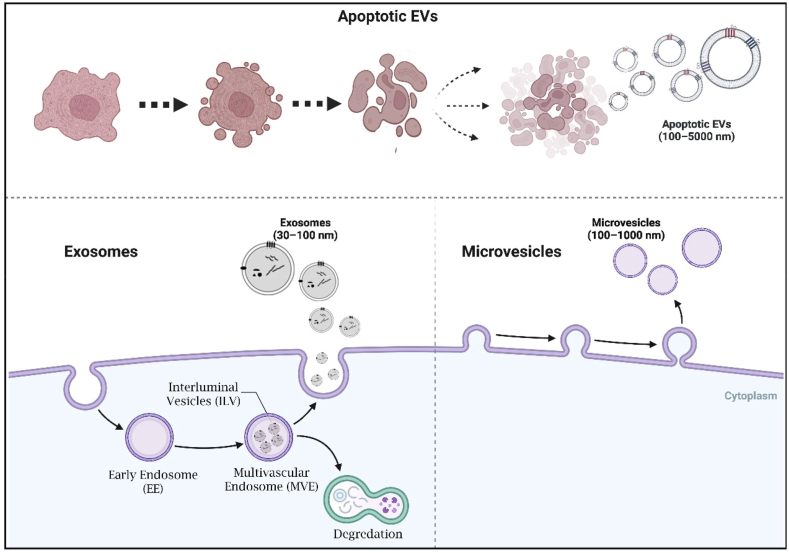
The biogenesis and categorization of EVs in cellular processes. Schematic illustration of EV biogenesis pathways, including exosomes, microvesicles, and apoptotic EVs. EV subtypes differ in size, origin, and release mechanism, reflecting distinct biological processes and functional implications. Exosomes (30–100 nm) are generated within MVEs through ILV formation. This is secreted upon MVE fusion with the plasma membrane. Furthermore, microvesicles (100–1000 nm) bud directly from the plasma membrane. Meanwhile, apoptotic EVs (100–5000 nm) are released from apoptotic cells during membrane blebbing and fragmentation.

EVs are naturally released by both prokaryotic and eukaryotic cells under physiological conditions and in response to disease-related stimuli^27^^,^^28^. The membrane-bound structures of EVs are characterized by their small size and are encapsulated with substances like lipids, nucleic acids, and proteins.^29^, ^30^, ^31^, ^32^ EVs mediate intercellular communication by delivering specific cargo to target cells while evading immune surveillance and resisting rapid degradation^7^^,^^33^^,^^34^. EVs are classified into three major groups based on their biogenesis and origin, namely: apoptotic EVs, microvesicles (microparticles or ectosomes), and exosomes (Fig. 1).^29^ Apoptotic EVs, also known as apoptotic bodies, are formed during the fragmentation of cells undergoing apoptosis, with a size ranging from 100 to 5000 nm^19^. Ectosomes originate from outward plasma membrane buds and typically measure between 1000 nm and 1 μm. Meanwhile, exosomes (30–150 nm) are formed through inward budding of the endosomal membrane, resulting in intraluminal vesicles (ILVs) within multivesicular endosomes (MVEs)^4^. During this process, endocytosed transmembrane proteins are reoriented while the cytoplasmic tails face the ILV lumen. The ectodomains are exposed outwardly, allowing these vesicles to participate in intercellular signaling^20^. When MVEs fuse with the plasma membrane, ILVs are released into the interstitial space as exosomes, which range from 30 to 150 nm^11^. The Endosomal Sorting Complexes Required for Transport (ESCRT) pathway is responsible for sorting cargo into ILVs and facilitating the release of MVBs from the endosomal membrane^12^^,^^29^.

### 2.2 EV content and structure

**Figure 2:**
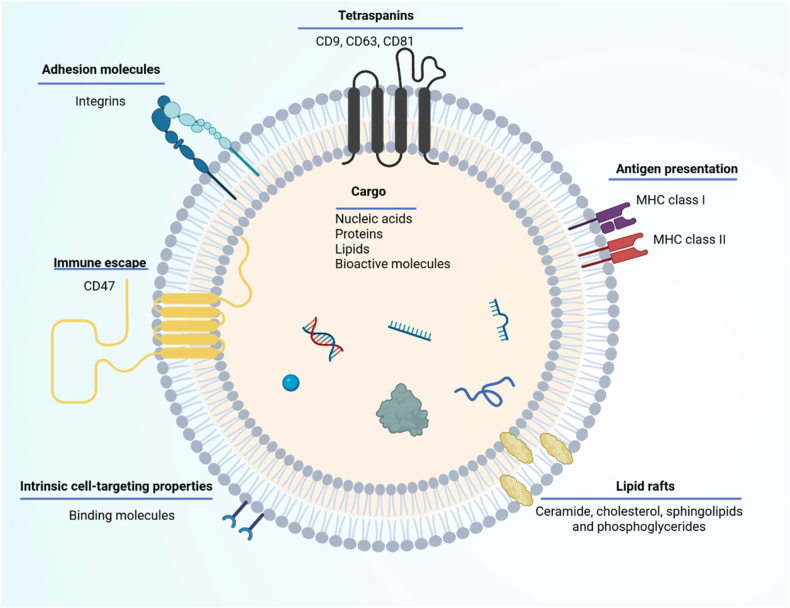
Structural components and molecular cargo of EVs. The illustration depicts EV architectural elements and molecular cargo like lipid rafts, surface antigens, and molecules (nucleic acids, lipids, and proteins). Tetraspanin proteins (CD63, CD81, and CD9) and adhesion molecules are highlighted for their roles in vesicle biogenesis and target-cell interaction.

EV structure consists of a membrane with proteins, lipids, and glycans, which enables them to bind to target cells (Fig. 2). The outer surface contains a wide variety of transmembrane and lipid-anchored proteins like integrins and tetraspanins (Fig. 2). EV proteins mirror the origin and physiological state of the donor cell, contributing to the signaling, enzymatic functions, and structural roles. For instance, tetraspanins (CD63, CD81, CD9) facilitate EV formation and target-cell interaction, while other protein cargoes like cytoskeletal proteins, heat shock proteins and growth factors contribute to EV structure and function (Fig. 2)^9^, ^13^, ^18^.

EVs are enclosed by a lipid bilayer membrane that supports their biological functions and enables intercellular communication^3^^,^^29^. This membrane also protects and facilitates interaction with target cells through the formation of a lipid mosaic. Additionally, lipid membranes like phospholipids, cholesterol, sphingolipids, and glycerolipids contribute to EV structure and function^14^. These lipids activate with metastasis-associated signaling pathways and transport “mobile rafts” that modulate target-cell behavior. For instance, ceramide, a sphingolipid on the EV surface, promotes lipid raft functionality while prostaglandins and lysophospholipids affect cell signaling during EV uptake^35^^,^^26^.

Glycans are essential for EV–cell binding *via* sugar–sugar and sugar–protein interactions^23^. Proteoglycans (PGs) are a common glycan structure that interacts with polybasic ligands, including apolipoproteins, viral capsid proteins, cationic lipids, nucleic acid‒peptide complexes, and polyamines^16^. The inherent ability of EVs to transport diverse molecular cargo, including nucleic acids, lipids, metabolites, and proteins, makes them valuable for delivering therapeutic agents^12^^,^^22^.

Nucleic acid encapsulation within EVs adds complexity to their cargo. For example, abundant microRNAs and other small non-coding RNAs in cells regulate gene expression post-transcriptionally^36^. EVs can transfer microRNAs from donor to recipient cells, thereby modulating gene expression and influencing various cellular processes^24^.

### 2.3 EV biological features

The internalization of EVs involves molecules from recipient cells, which activate signaling pathways leading to a pathophysiological cascade change within the cells. In addition to their intricate cargo composition, EVs contribute to intercellular communication through specific biological features such as diversity, surface markers and immunomodulatory potential^37^. Different EV subpopulations arise from distinct biogenesis processes and cellular sources^38^. For example, microvesicles bud from the plasma membrane, whereas exosomes originate from the endosomal system. Surface markers like tetraspanins, integrins, and cell-specific proteins distinguish the different EV subtypes and enhance cell-to-cell communication^39^. EVs also modulate immune cell activation and polarization by shuttling immunoregulatory molecules, cytokines and antigen-presenting molecules^40^.

## 3 Sources of EVs

EVs originate from different sources due to their unique bioactive components in cells. These cellular sources include cancer cells, immune system cells, stem cells, blood cells, endothelial cells, and adipose tissue cells^41^. Additionally, EVs can be collected from various biological fluids, including urine, serum, plasma, saliva, tears, breast milk, gastric secretions, malignant pleural effusion, prostatic fluid, amniotic fluid, cerebrospinal fluid, joint fluid, and bronchoalveolar lavage (BAL) fluid^42^. The choice of source depends on factors such as the mode of action, the cargo or bioactive molecules carried by the EVs, and their interactions within the TME^43^.

### 3.1 Cells

The choice of cell type is crucial when selecting a source for EVs, especially for drug delivery. Different cell types possess distinct characteristics, such as membrane rigidity and surface markers from donor cells, when a specific cell type is selected^41^.

#### 3.1.1 Cancer cells

In cancer therapy, cancer cells produce exosomes by overexpressing the Rab27A and Rab27B proteins, rendering these vesicles valuable for delivering drugs^44^. For instance, immune suppressive and evasive proteins like programmed death-ligand 1 (PD-L1) and transforming growth factor beta (TGF-*β*) are expressed by tumor-derived EVs^45^^,^^46^. Studies have shown that exosomes secreted by breast cancer cells express high levels of PD-L1 in response to dose-dependent treatment with TGF-*β*. However, low TGF-*β* levels attenuate exosome release, leading to reduced tumor burden with enhanced T-cell cytotoxicity^47^. Additionally, patient-derived exosomes after treatment with Radium-223 (^223^Ra) were found to express PD-L1. Moreover, combining^223^Ra with immune checkpoint therapy (ICT) had remarkable potency in the Myc-CaP mouse model as compared with either treatment alone^48^. These findings indicate that inhibiting PD-L1 or TGF-*β* signaling in tumor-derived EVs may enhance immune responses and boost the effectiveness of immune checkpoint therapies.

#### 3.1.2 Immune cells

Immune cells are sources of EVs because of their capacity to stimulate a potent antitumor immune response^41^^,^^49^. EVs from immune cells can be derived from various types of immune cells, including dendritic cells, natural killer (NK) cells, macrophages, etc^50^, ^51^, ^52^. Such EVs enhance immune responses. For instance, exosomes from macrophages help mediate interactions with cancer cells, leading to reduced tumor progression^53^^,^^54^. Dendritic cell-derived exosomes (dexosomes) have been evaluated in clinical trials. In a phase I study of stage III/IV melanoma, patients were vaccinated with autologous exosomes loaded with MAGE-3 peptides. The treatment was well-tolerated, with no toxicity and no maximum tolerated dose reached^55^. Additionally, some patients showed prolonged disease stability and immune activation in a phase I trial carried out on advanced NSCLC patients expressing HLA-A2+ and MAGE-A3/A4 tumor antigens^56^. The utilization of first-generation dexosomes with or without IFN-*γ* loaded with MHC class I/II-restricted tumor antigens, enhanced NK cell-mediated antitumor responses in phase I/II trials^57^. In summary, immune cell-derived exosomes hold the potential to enhance antitumor immunity.

#### 3.1.3 Mesenchymal stem cells

Mesenchymal stem cell (MSC)-derived EVs could be a cellular therapy due to their minimal activation of immune responses and reduced risk of promoting cancer^58^, ^59^, ^60^, ^61^. For instance, systemic delivering miR-122 *via* adipose tissue-derived MSC (AD-MSC) exosomes sensitizes hepatocellular carcinoma (HCC) cells to chemotherapeutic agents^62^. Additionally, AD-MSC-derived EVs enhance breast cancer sensitivity to cisplatin through miR-1236 delivery. This microRNA downregulates SLC9A1 and inhibits the Wnt/*β*-catenin pathway^63^. Ongoing Phase 1 trial (NCT03608631) at The University of Texas MD Anderson Cancer Center, which is evaluating the safety and optimal dosage of MSC-derived exosomes loaded with KRAS^G12D^ siRNA. This treatment is delivered intravenously to patients with metastatic pancreatic cancer harboring the KRAS^G12D^ mutation^64^. In conclusion, MSC-derived EVs enhance cancer treatment outcomes by offering efficient targeted delivery of therapeutic molecules to tumor sites.

### 3.2 Body fluids

Body fluids such as blood, cerebrospinal fluid (CSF), saliva, urine, serous fluid, and amniotic fluid can serve as sources of EVs^65^. For example, plasma-derived EVs offer a minimally invasive method for cancer diagnosis and monitoring^66^^,^^67^. These circulating EVs in serum or saliva-derived EVs carry tumor-specific biomolecules from tumor cells, including DNA, RNA, proteins, and lipids^68^. This approach supports cancer diagnosis and prognosis by analyzing serum-derived EVs containing circulating tumor DNA (ctDNA) and miRNAs, or by analyzing molecules from oral or systemic cancers^67^. For instance, red blood cell-derived EVs can effectively deliver therapeutic RNA molecules, including siRNAs, antisense oligonucleotides (ASOs), and clustered regularly interspaced short palindromic repeats CRISPR/Cas9 guide RNAs^22^. Saliva-derived EVs have also been exploited to develop diagnostic tests for oral cancer by identifying mutations and gene expression patterns linked to the disease^69^. Previous studies have shown that red blood cell-derived EVs bearing triantennary *N*-acetyl galactosamine sequences (GalNac) could provide protective effects in hepatocellular carcinoma, acute liver failure, and non-alcoholic fatty liver disease^70^. Furthermore, studies have shown that EVs loaded with DOX or sorafenib could effectively treat localized HCC while reducing systemic toxicity^71^. In another study, EVs isolated from ascitic fluid of colon cancer patients, combined with granulocyte-macrophage colony-stimulating factor, are shown to boost cytotoxic T lymphocyte responses against specific tumor antigens^72^. In summary, EVs derived from body fluids represent a promising avenue for cancer treatment, offering targeted delivery, stability, and versatility in cargo packaging.

### 3.3 Milk

Milk-derived EVs have effective absorption after ingestion due to increased IgG interaction with the Fc receptor in the gastrointestinal tract^73^^,^^74^. In cancer immunotherapy, milk-derived exosomes are utilized to enhance the immune response against cancer. For instance, studies have shown that EVs obtained from bovine milk can transport drugs such as paclitaxel, docetaxel, and DOX, improving their absorption and efficacy in cancer treatment^75^, ^76^, ^77^, ^78^. Studies have also demonstrated that oral delivery of celastrol (CEL) with milk-derived exosomes can suppress breast cancer cell growth in mice, enhancing both drug stability and cellular uptake^79^^,^^80^. Furthermore, engineered milk-derived exosomes loaded with berry anthocyanidins enhance the stability and availability of Anthos, resulting in effective ovarian cancer treatment^81^. Milk-derived exosomes loaded with Texas Red-labeled siRNA have also been shown to efficiently deliver siRNA into lung cancer cells^82^. Additionally, milk-derived exosomes have effectively delivered Bcl-2–siRNA to the cell membrane, resulting in reduced invasiveness and migration of pancreatic cancer cells^83^. This indicates that milk-derived exosomes could play a promising role in cancer therapy due to their natural biocompatibility, stability, and ability to deliver therapeutic agents.

### 3.4 Plants

Plant-derived EVs are from edible plants. They are produced in large quantities due to its resistance to gastric proteolytic and pancreatic enzymes, which contributes to high oral tolerance^84^, ^85^, ^86^, ^87^. Previous studies have shown that modified ginger-derived nanovectors (GDNVs) conjugated with folic acid and loaded with DOX inhibited tumor growth in colon cancer^85^^,^^88^. Furthermore, nanocarriers derived from grapefruit improved the efficacy of drug delivery and specifically targeted inflamed tumors^89^. Ginger-derived EVs encapsulated with bioactive compounds like gingerol and shogaol can effectively transport them to cancer cells, therebysuppressing tumor growth^90^^,^^91^.

### 3.5 Bacteria

Outer membrane vesicles (OMVs) from bacteria can carry immune-activating molecules that boost the immune response^41^^,^^92^. Previous studies have shown that vesicles from *pneumococcal* strains’ outer membrane can effectively deliver DOX to NSCLC cells, resulting in increased antitumor efficacy through immune system activation^92^. Additionally, bacterial EVs serve as targeted delivery vehicles for cancer therapy^93^. For instance, previous studies have shown that vesicles from the outer membrane of bacteria with high expression of epidermal growth factor (EGF) can deliver DOX and idarubicin to tumor cells, leading to reduced tumor growth in mice^94^. These findings highlight that bacteria-derived EVs could be an immunotherapeutic tool against cancer.

## 4 Strategies for EV functionalization in cancer

**Figure 3:**
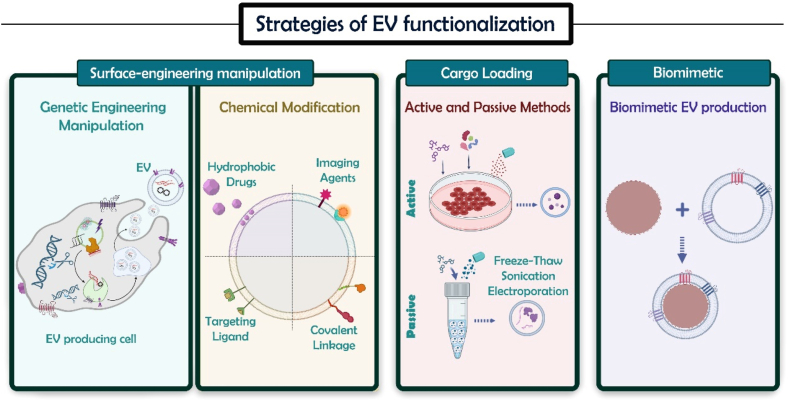
Overview of various strategies for EV functionalization in cancer treatment. Strategies encompass surface engineering manipulation (genetic engineering and chemical modification), cargo loading (active andpassive methods), and biomimetic EV production. These approaches aim to increase the therapeutic potential of EVs by improving their targetability, stability, and cargo delivery capabilities.

In cancer treatment, EVs improve specificity and efficacy through precise drug delivery and cargo transport (Table 1)^95^. EV modifications are classified as direct or indirect, with direct methods being more straightforward and versatile as compared to genetic engineering techniques (Fig. 3)^96^.

**Table 1:** Encapsulated EVs for cancer treatment.

Enclosed agent	Agent	EVs source	Loading methodology	Cancer type	Outcome	Ref.
Drugs	DOX	Dendritic cells	Electropermeabilization	Breast cancer	Suppressed tumorigenesis	120
	DOX	MSCs	Electropermeabilization	Colon carcinoma	Impede cancer cell growth	131
	DOX	Colorectal cancer cells	Agitation and dialysis	Colon carcinoma	Reduced cancer cell proliferation	132
	DOX	MSCs or Cancer-associated fibroblasts (CAFs)	Extrusion	Ovary cancer	Cytotoxic effect on the ovarian cancer cells	133
	DOX	Citrus fruit	Incubation	Malignant glioma	Effect on the immunosuppressive tumor milieu	134
	Paclitaxel (PTX)	MSCs	Extrusion	Breast cancer	Suppressed cancer cell viability	135
	PTX	Macrophage	Acoustic treatment	Lung cancer	High cytotoxicity and reduced cancer cell proliferation	136
	PTX	Macrophage	Sonication	Breast cancer	Reduced tumor growth	137
	Melanin and paclitaxel albumin	Breast cancer cell line	Incubation and Extrusion	Breast cancer	High infiltration of CD8+ T-cells in mouse tumor tissues	138
	TXL	MSCs	Extrusion	Lung, ovarian, and breast cancer	Reduced cancer cell growth	139
	CDDP	Hepatocarcinoma cells and ovarian cancer cells	Diffusion	Ovarian cancer and hepatocarcinoma	Suppressed cancer cell proliferation	140
	Erastin	Human lung cancer cells	Sonication	Breast cancer	Impede cancer cell proliferation	141
	Atorvastatin	Human endometrial stem cells	Freeze‒thaw cycles, sonication, and diffusion	Glioblastoma	Elevated level of apoptosis in cancer cells	142
	5-FU	Colorectal cancer cells	Electropermeabilization	Colon cancer	Suppressed tumor growth	143
	Staurosporine	Human umbilical cord mesenchymal stem cells (hUC-MSCs)	Incubation	Esophageal squamous cell carcinoma	Inhibit ABCA1 transporter *via* elevated radiosensitivity in cancer cells	123
	GEM	MSCs, human cancerous lymphoma, monocytic leukemia, and pancreatic cancer cells	Incubation	Pancreatic cancer	Reduced cytotoxicity	144
	Curcumin	T lymphoblast	Agitation	Glioblastoma	Increase apoptosis	145
	Curcumin	Bovine milk	Agitation	Lung, cervical, and breast cancer	Hinders cancer cell proliferation	146
Proteins	Survivin-T34A	Melanoma cells	Gene transfer	Pancreatic adenocarcinoma	Promote apoptosis and drug sensitivity	147
	TRP2	Serum	Electropermeabilization	Different cancer types	Increased accumulation in lymph nodes	148
	SIRPα	Human embryonic kidney 293 cells (HEK-293)	Gene transfer	Colon carcinoma	Enhanced cancer cell phagocytosis	149
	Cyclic GMP-AMP synthase (cGAS)	Human embryonic kidney 293 cells	Gene transfer	Colon adenocarcinoma	Suppressed tumor growth	150
	hMUC1	Colon cancer cells and breast cancer cells	Gene transfer	Colon carcinoma	Enhanced immune response	151
Nucleic acids	miRNA	miR-567	Breast cancer cells	Electropermeabilization	Reduced cell proliferation	152
		Let-7a miRNA	Human embryonic kidney 293 cells	Electropermeabilization	Reduced cell proliferation	153
		miR-379	MSCs	Electropermeabilization	Reduced cell proliferation	154
		miR-146b	MSCs	Electropermeabilization	Suppressed glioblastoma	155
		miRNA-122	AD-MSCs	Electropermeabilization	Impede hepatocellular carcinoma progression	156
		miRNA-21	Human embryonic kidney 293 cells	Electropermeabilization	Suppressed brain carcinoma	157
	CircRNA	circ-0051443	Hepatocellular carcinoma cells	Overexpression	Impede hepatocellular carcinoma	158
		circIFNGR2	CAFs	Overexpression	Suppressed ovarian cancer progression	159
		circSTAU2	Gastric cancer cells	Overexpression	Reduce gastric cancer progression	160
	siRNA	HER2-siRNA	HEK293T and MCF-7 cells	Sonication	Impede breast cancer progression	161
		BCL6- siRNA	Dendritic cells (DCs)	Electropermeabilization	Suppressed Large B-cell lymphoma progression	162
		c-Met siRNA	Human embryonic kidney 293 cells	Gene transfer	Reduce gastric cancer progression	162
	mRNA	*IL-*12 mRNA	Human bone marrow-derived mesenchymal stem cells (hBM-MSCs)	Electropermeabilization	Reduce lung cancer progression	163

### 4.1 Surface engineering manipulation

EV modification involves altering the surface membrane, which is less cumbersome and highly appealing for screening new targeting ligands, like biotin-conjugated ligands or visualizing agents attached to the EV surface^97^. EV surface engineering modulates the TME by manipulating antibodies, peptides, and proteins, resulting in enhanced target specificity^98^. In cancer immunotherapy, the manipulation of membrane proteins and lipids is shown to enhance cell-specific delivery^99^. This can be achieved through genetic/biological alterations or chemical modifications.

#### 4.1.1 Genetic engineering manipulation

Genetic manipulation enables tissue-specific EV targeting, and therebycell-specific delivery of therapeutic molecules^97^. This approach involves engineering donor cells with plasmids encoding ligand- or peptide-fused membrane proteins. As a result, secreted exosomes display the targeting ligands on their surface^99^. Previous studies have shown that EVs from the streptavidin-biotin system require the isolation of EVs from modified donor cells with DMPE-phospholipids conjugated to streptavidin through a polyethylene glycol (PEG) linker^100^. Additionally, reports have shown that LAMP-2B is an exosomal surface protein used to effectively deliver DOX to integrin-positive breast tumor cells, resulting in impeded cancer growth^101^. Genetic modification has also been employed to display surface antigens or deliver immune checkpoint inhibitors for cancer therapy.

#### 4.1.2 Chemical modification

Chemical modification uses reagents to add moieties such as proteins, immunoglobulins, fluorescent markers, and other immune-regulating substances to the TME^102^. This functionalized approach conjugates large biomolecules on exosomes, leading to improved cancer therapy^98^^,^^99^. Chemical conjugation of EVs can be covalent or noncovalent. The covalent methods involve directly attaching ligands to the EV surface, while noncovalent methods rely on weaker interactions like ionic bonds, hydrophobic forces, or ligand–receptor binding^98^. Another approach is the click chemistry that modifies amine group to alkyne group, enabling the conjugation of alkyne-tagged exosomal proteins with azide-based reagents *via* copper-catalyzed azide‒alkyne cycloaddition (CuAAC)^99^. For example, in glioma-targeting RGE peptide (RGERPPR) modification, exosomes are linked through a cycloaddition reaction with sulfonyl azide^103^. Incorporating amphipathic molecules into the lipid bilayer of exosomes is also an approach and this involves combining the tumor-targeting ligand folate with PEG-*b*-1,2–distearoyl-*sn*-glycero-3–phosphoethanolamine (PEG-*b*-DSPE), resulting in improved cancer treatment outcomes^99^.

### 4.2 Cargo loading or physical methods

The different approaches of cargo loading or physical methods are electropermeabilization, incubation, sonication, extrusion, freeze–thaw cycles, and endogenous loading (Table 1). These techniques create surface pores that interact with or bind to accessible membrane lipids or proteins^98^. The cryocycling approach involves the formation of ice crystals *via* the development of hybrid exosome–liposome-fused vesicles for drug delivery^104^. Meanwhile, electropermeabilization uses an electric field to temporarily disrupt the cell membrane, allowing external substances to enter EVs and enabling targeted delivery^98^. The EV cargo loading incubation strategy offers a promising approach for preparing EV drug delivery systems^78^. Finally, microfluidic technology has been employed to modify exosome surfaces by incorporating tumor antigenic peptides^105^.

### 4.3 Biomimetic EV production

Therapeutic cargo delivery can be carried out by using polymeric nanocarriers (PNCs), NPs and bionic nanomedicine technologies to target cancer cells^106^. For instance, studies have demonstrated that coating EVs with high-density nanoraspberries can effectively inhibit tumor metastasis^107^. Furthermore, vesicles derived from breast cancer cells have demonstrated enhanced cargo-loading abilities and a longer circulation lifespan than synthetic liposomes do^108^. The use of chimeric peptide-modified exosomes has been engineered into nanosized vesicles to transport photosensitizers to the plasma membrane and nucleus, enhancing the efficacy of combined photodynamic therapy (PDT)^104^. PDT using engineered exosomes involves three key steps: (1) generating reactive oxygen species (ROS) to induce ferroptosis in tumor cells, (2) releasing therapeutic agents triggered by ROS and (3) applying a two-stage light treatment to destroy the plasma membrane and nucleus of tumor cells. Additionally, coating urokinase-type plasminogen activator-engineered extracellular vesicles (uPA-eEVs) with PNCs improves tumor cell targeting, producing an antitumor effect in 4T1 tumor-bearing mice^108^.

## 5 Gene editing strategies for engineering EVs in cancer therapy

**Figure 4:**
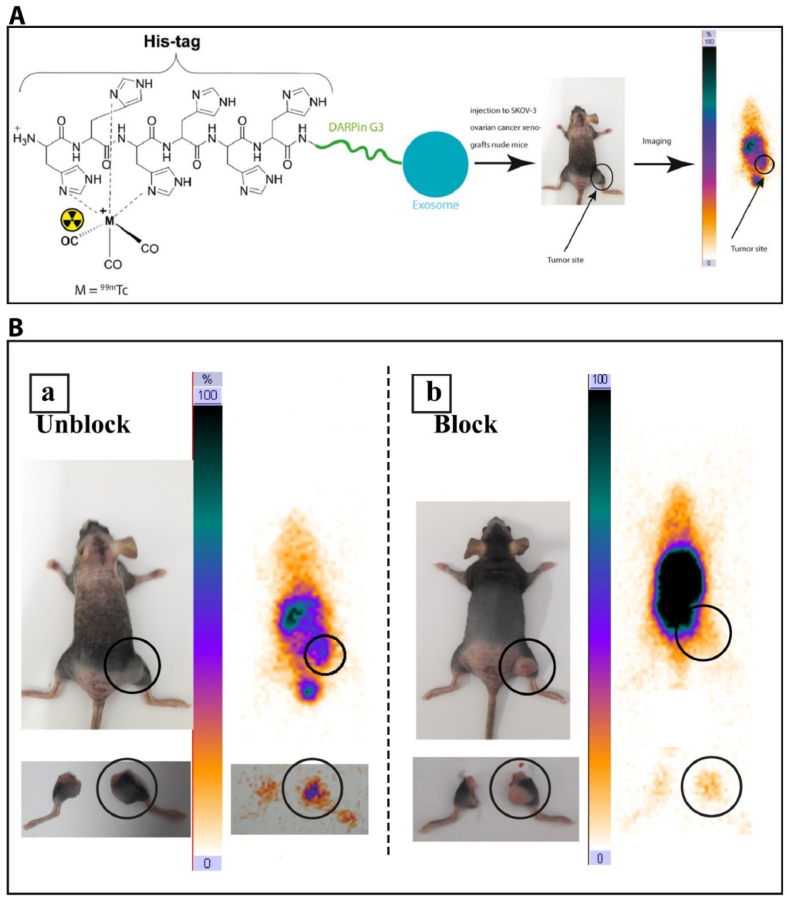
HER2-targeted tumor imaging using radiolabeled exosomes. (A) Successful radiolabeling of genetically modified tumor-derived exosomes with ^99m^Tc as demonstrated by Molavipordanjani et al.^184^. In SKOV-3 tumor-bearing nude mice with high HER2 expression demonstrated a high accumulation of exosomes. This highlights the potential of ^99m^Tc-radiolabeled exosomes as a targeted imaging modality for tumors overexpressing HER2 receptors. (B) The whole-body planar imaging of SKOV-3 ovarian cancer xenograft nude mice treated with trastuzumab antibody for HER2 receptor saturation 1 h before ^99m^Tc-exosome injection at 4 h in a blocked mouse^184^. Reprinted with permission from Ref. 184. Copyright ©2020, Elsevier.

Gene editing of parent cells creates customized EVs with improved functionality and targeted delivery capabilities (Table 2)^109^. CRISPR/Cas9 technology can be used to insert, delete, or alter specific genes in parent cells, which impacts the molecular content and surface markers of EVs^109^, ^110^, ^111^, ^112^. This involves precise genomic modifications to parent cells, facilitating the presentation of targeting ligands, immunomodulatory molecules, or other functional components on the resulting EVs^109^. Genetic modification of parent cells improved engineered EVs to target specific cells, enhance immune regulation, increase drug loading, and create more uniform EV populations with consistent characteristics^109^. Additionally, this method enables the production of uniform EV populations with consistent properties, improving the efficacy of EV-mediated treatments^109^^,^^110^. For example, cancer-derived exosomes (CDExs) have been used as precise delivery mechanisms for CRISPR/Cas9 to target cancer cells without sparing normal cells (Fig. 4)^113^.

**Table 2:** Gene editing strategies for engineering EVs in cancer therapy.

Gene editing approache	Interacting genes and proteins/vector	EV origin	Loading methodology	Cancer type	Function	Ref.
CRISPR/Cas9	The stop element/Cas9 ribonucleoprotein	HEK293T	Transfection	Lung cancer	Engineered exosomes emit discernible red fluorescence	172
CRISPR/Cas9	Kras^G12D^/Plasmid	HEK293T	Transfection	Pancreatic cancer	Suppress cell proliferation and tumor growth	173
CRISPR/Cas9	WNT10B/Cas9 ribonucleoprotein	HEK293T	Freeze‒thaw, sonication	Hepatocellular carcinoma	Decreased cell viability and impeded tumor growth	174
CRISPR/Cas9	MYC/Plasmid	HEK293T	Electropermeabilization	B-cell malignancies	Reduced tumor growth and triggered cell apoptosis	175
CRISPR/Cas9	IQGAP1/Plasmid	HEK293T	Electropermeabilization	Hepatocellular carcinoma	Promoted cell apoptosis	176
CRISPR/Cas9	miR-125b/sgRNA + Cas9 mRNA	Red blood cell	Electropermeabilization	Acute myeloid leukemia	Repressed expression of miR-125b	33
CRISPR/Cas9	PARP1/Plasmid	SKOV3	Electropermeabilization	Ovarian cancer	Suppressed tumor growth, apoptosis, and cell proliferation	169
CRISPR/Cas9	Stop-fluorescent protein elements/sgRNA + Cas9 protein	melanoma	Transfection	Tumor cell	Triggered organ-specific metastasis of melanoma	175
CRISPR/Cas9	Aptamer/Cas12a proteins	Tumor	Transfection	Nasopharyngeal carcinoma	Improved prognosis and diagnosis for nasopharyngeal carcinoma	177
CRISPR/Cas9	Aptamer/Cas9 proteins	Breast cancer line	Transfection	Breast cancer	Improve the diagnosis of breast Cancer	178
CRISPR/Cas9	IQGAP1, FOXM1/Cas9 molecules and dual sgRNA plasmid	LC9-293 and HN3LC9-293	Electropermeabilization	Hepatocellular carcinoma	A decline in the CD133+ population, which supports liver cancer cells' stemness	179
CRISPR/Cas9	Angiopep-2 and the *trans*-activator of transcription (TAT) peptide/plasmids or Cas9/sgRNA	N/A	Electropermeabilization, freeze-thawing, and sonication	Glioblastoma	Enhances the effectiveness of treatment by eliminating therapeutic resistance and sensitizing GBM cells to radiation.	180
CRISPR/Cas9	pLenti-CRISPR-V2 along with pMDLg/pRRE, pRSV-Rev and pCMV-VSV-G virus packaging plasmid	Epithelial cell	Transfection	Hepatocellular carcinoma	Enhances anticancer effect in HepG2 xenografts	181
Gene transfection	7D12 hCD47 decorations, miR-204-5p/	HEK293T	Transfection	EGFR + tumors	Boost tumor-suppressive effects in EGFR-positive tumor cells	182

Furthermore, exosomes can serve as carriers for artificial circular RNAs (acRNAs) and CRISPR/Cas9 in bladder cancer gene therapy. Studies have shown that cell-derived vesicles can deliver acRNAs to selectively silence cancer-related genes, leading to reduced tumor growth and metastasis in animal models^114^. Despite their potential benefits, the use of CRISPR-engineered EVs faces challenges such as off-target effects, immune reactions, and scalability issues^109^^,^^110^. Additional optimizations are needed to address these challenges and fully leverage CRISPR-engineered EVs for targeted delivery and precision medicine.

## 6 Integrating cancer treatment and imaging in engineered EVs for imaging-guided therapy

*In vivo* imaging and tracking of EVs are essential for evaluating delivery efficiency, understanding their biological functions, and monitoring uptake and persistence in the body (Table 3)^115^^,^^116^. Real-time imaging is essential in therapy guidance, enabling accurate tracking of treatment delivery and metabolism for more precise interventions^117^. Furthermore, post-therapy lesion imaging evaluates therapeutic efficacy and detects potential damage to adjacent healthy tissues^118^.

**Table 3:** Different EV-based imaging labeling methods for imaging-guided cancer therapy.

EV source	EV subtype	Cancer type	Engineered strategy	Imaging agent	Application	Ref.
Adipose tissue-derived MSC (AD-MSC)	Exosomes	Various Cancers	Ultrasmall superparamagnetic iron oxide (USPIO) labeling for MRI tracking	USPIO nanoparticles	Non-invasive tracking of exosome homing and therapeutic potential	194
Mesenchymal stem Cells (MSCs)	Exosomes	Glioblastoma (GBM)	Angiopep-2 peptide-modified exosomes with magnetic nanoparticles (MNPs) for BBB penetration and tumor targeting	MNPs	Ferroptosis therapy in GBM	195
Melanoma Cells	Exosomes	Melanoma	Superparamagnetic iron oxide nanoparticles (SPIONs)-loaded exosomes *via* electroporation	SPIONs	MRI visualization of exosome homing in lymph nodes	196
Breast Cancer Cells	Exosomes	Breast Cancer	Magnetic graphene oxide nanoparticles (MGONs) functionalized with CD63 aptamers	Fe3O4@SiO2 nanoparticles	Rapid exosome capture and fluorescence-based quantification	197
Human Placental MSCs	Exosomes	Breast Cancer	Exosomes loaded with Dox and modified with carboxylated Fe3O4 nanoparticles	Carboxylated Fe3O4 nanoparticles	Targeted drug delivery and tumor growth suppression	198
Reticulocytes	Exosomes	Various Cancers	Superparamagnetic nanoparticles (SMNC-EXOs) for magnetic targeting	Superparamagnetic nanoparticles	Enhanced tumor targeting and drug delivery	199
Human umbilical Cord MSCs	Exosomes	Osteosarcoma	Exosomes labeled with gadolinium (GdL) for MRI or NIR dyes for fluorescence tracking	GdL or NIR dyes	Tumor targeting and therapeutic delivery	200
Breast Cancer Cells	Exosomes	Breast Cancer	Magnetic SERS platform for exosome isolation and analysis	Magnetic nanoparticles	Real-time liquid biopsy for cancer detection	17
Hepatocellular Carcinoma	Exosomes	Hepatocellular Carcinoma	Fluorescent biosensor with MNPs and horseradish peroxidase (HRP) for exosome detection	MNPs and HRP	Early cancer diagnosis using exosomes as biomarkers	20
Hepatic Carcinoma Cells	Exosomes	Hepatic Carcinoma	Fluorometric assay with MNPs and hybridization chain reaction (HCR)	MNPs	Sensitive detection of cancer-related exosomes	18
Pancreatic Cancer Cells	Exosomes	Pancreatic Cancer	Microfluidic device with Fe3O4 nanoparticles for exosome isolation	Fe3O4 nanoparticles	Early cancer diagnosis and monitoring	21
Melanoma Cells	Exosomes	Melanoma	Self-calibrated magnetic aptamer sensor (sCMAS) for PD-L1 detection	Tb-based nanoparticles (TbNps)	Non-invasive melanoma diagnosis and monitoring	32
Breast Cancer Cells	Exosomes	Breast Cancer	Aptamer-functionalized magnetic composite (FPTA) for PD-L1-positive exosome isolation	Magnetic nanoparticles	Cancer diagnosis and immunotherapy monitoring	201
Breast Cancer Cells	Exosomes	Breast Cancer	Magneto-mediated electrochemical sensor for exosomal protein analysis	Magnetic beads and silica nanoparticles	Clinical diagnosis and prognosis of breast cancer	37
Breast Cancer Cells	Exosomes	Breast Cancer	Dual-targeting nanovesicle system with MNPs for ferroptosis therapy	Superparamagnetic nanoparticles	Ferroptosis therapy in migratory breast cancer	38
Breast Cancer Cells	Exosomes	Breast Cancer	CRISPR/Cas12a-based electrochemical aptasensor for exosome detection	MNPs	Early breast cancer diagnosis	27
Oral squamous Cell Carcinoma	Exosomes	Oral squamous Cell Carcinoma	Cholesterol-EpCAM aptamer-mediated proximity ligation assay (PLA) with MNAzyme	Magnetic beads and terbium (Tb) tags	Early cancer diagnosis	28
Breast Cancer Cells	Exosomes	Breast Cancer	Superparamagnetic iron oxide (SPIO)-labeled FeEVs for brain metastasis imaging	SPIO	Targeting and imaging brain metastases	20
HEK-293T Cells	Exosomes	Breast Cancer	Dual pH/US-sensitive EVs loaded with ICG, PTX, and SBC	Indocyanine green (ICG)	Photoacoustic imaging-guided chemo- sonodynamic therapy (SDT)	202
Various Cell types	Small EVs (sEVs)	Various Cancers	Metabolic labeling with Bodipy FL C16	Bodipy FL C16	Visualization and tracking of sEVs in cancer treatment	203
Various Cell types	Exosomes	Bladder Cancer	Dual-targeting peptide/exosome/cobalt sulfide QDs	Cobalt sulfide QDs	Targeted delivery and synergistic action of chemodynamic and photothermal therapy (PTT)	204
B16-BL6 Cells	Exosomes	Melanoma	GLuc-lactadherin labeling	Gaussian luciferase (GLuc)	*In vivo* tracking of exosome biodistribution and clearance	205
Macrophage-derived Cells	Exosome-mimetic nanovesicles (ENVs)	Various Cancers	Radiolabeling with 99 mTc-HMPAO	99 mTc-HMPAO	Preferential uptake in the liver and tumor imaging	206
Tumor-derived Cells	Exosomes	Ovarian Cancer	Radiolabeling with^99m^Tc	^99m^Tc	HER2-targeted tumor imaging in SKOV-3 tumor-bearing mice	170
Melanoma Cells	Exosomes	Melanoma	Radiolabeling with^111^In	^111^In	Improved efficiency and stability of radiolabeling in melanoma imaging	207
Various Cell types	Exosomes	Melanoma	SPION-loaded exosomes	Superparamagnetic iron oxide nanoparticles	MRI tracking of exosomes in lymph nodes	196
M1 macrophages	Exosomes	Breast Cancer	Combination therapy with CPPO, Ce6, and Dox-EMCH	Photosensitizer (Ce6)	Synergistic trimodal anticancer therapy (immunotherapy, PDT, chemotherapy)	208
Various Cell types	Exosomes	Various Cancers	Combination therapy with DOX, magnetic NPs, and molecular beacons	Magnetic nanoparticles	Chemotherapy, gene therapy, and PTT	209
Various Cell types	Exosomes	Various Cancers	SDTwith ICG, PTX, and SBC	ICG	Dual stimuli-responsive sonosensitizers for combination therapy	210
Various Cell types	Exosomes	Various Cancers	Polydopamine (PDA)-based PTT with DOX and miR-21	PDA	Multimodal therapeutic platform for cancer treatment	211

EVs, extracellular vesicles; ASCs, adipose stem cells; MSCs, mesenchymal stem cells; GBM, glioblastoma; MNPs, magnetic nanoparticles; BBB, blood-brain barrier; SPIONs, superparamagnetic iron oxide nanoparticles; MGONs, magnetic graphene oxide nanoparticles; Fe_3_O_4_@SiO2, iron oxide silica core-shell nanoparticles; Dox, doxorubicin; Fe_3_O_4_, iron oxide; SMNC-EXOs, superparamagnetic nanoparticle-engineered exosomes; GdL, gadolinium-labeled; NIR, near-infrared; HRP, horseradish peroxidase; HCR, hybridization chain reaction; sCMAS, self-calibrated magnetic aptamer sensor; TbNps, terbium-based nanoparticles; SPIO, superparamagnetic iron oxide; PD-L1, programmed death-ligand 1; PLA, proximity ligation assay; MNAzyme, magnetic nucleic acid enzyme; FPTA, functionalized photothermal agent; PTX, paclitaxel; SBC, sulforaphane-based compound; Bodipy FL C16, boron-dipyrromethene FL C16; QDs, quantum dots; Gluc, gaussian luciferase; ENVs, exosome-mimetic nanovesicles; ^99m^Tc-HMPAO, Technetium-99m hexamethylpropyleneamine oxime; ^99m^Tc, Technetium-99m; ^111^In, Indium-111; CPPO, 2-(6-chlorobenzo[d]thiazol-2-yl)-5-(4-methoxyphenyl)-1,3,4-oxadiazole; Ce6, Chlorin e6; SDT, sonodynamic therapy; PDA, polydopamine; miR-21, microRNA-21; PTT, photothermal therapy; PDT, photodynamic therapy; HER2, human epidermal growth factor receptor 2; SKOV-3, ovarian cancer cell line; EpCAM, epithelial cell adhesion molecule; USPIO, ultrasmall superparamagnetic iron oxide nanoparticles.

**Figure 5:**
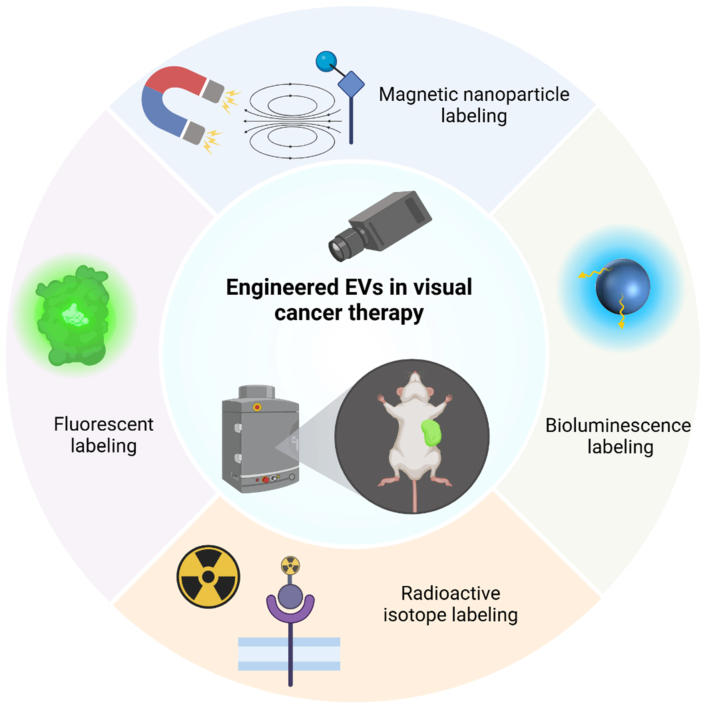
Engineered EVs in imaging-guided cancer therapy. Overview of labeling techniques, like magnetic nanoparticles, fluorescence, radioactive isotopes, bioluminescence, and quantum dots, to track engineered EVs in image-guided cancer therapy. This offers specific benefits for visualizing EV delivery, distribution, and therapeutic efficacy.

Exosome biodistribution by using imaging agents like fluorescent dyes or magnetic NPs serves as a tool in imaging-guided therapy^119^^,^^120^. This can be either internal or external labeling. The externally labeled exosomes from cell culture are commonly tagged with membrane dyes like DiI, PKH67, or PKH26^121^^,^^122^ while the internally labeled exosomes are generated through the genetic modification of exosome-producing cells^123^. The various techniques used to label EVs for imaging are illustrated in Fig. 5. Previous studies have demonstrated that autologous EVs from cancer patients loaded with near-infrared dye indocyanine green (ICG) could be used for intraoperative visualization of tumor margins^124^. Preclinical studies also demonstrated that labeling enhances selective tumor accumulation, high-contrast imaging, and a favorable safety profile. This also had scalable isolation, resulting in potential clinical translation for surgical precision^124^.

### 6.1 Fluorescent labeling

**Figure 6:**
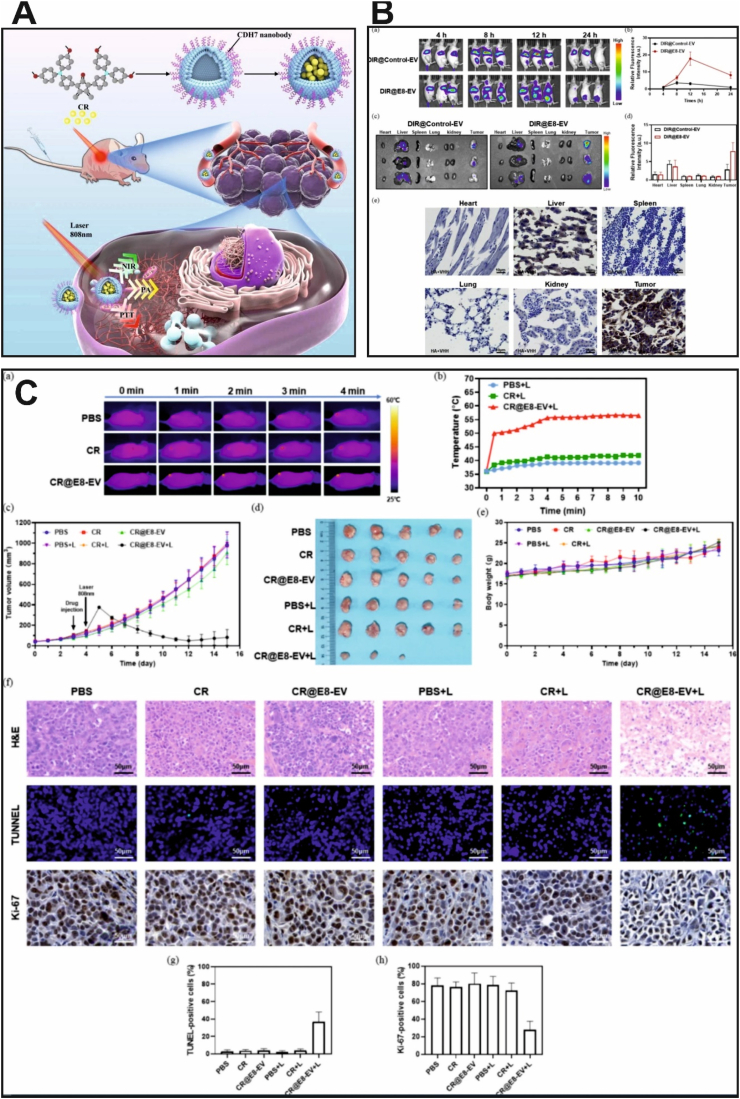
Imaging-guided photothermal therapy (PTT) using engineered EVs. (A) This shows how HEK-293 cells are designed to selectively target CDH7-positive cancer cells for photothermal ablation therapy in gastric cancer by using engineered EVs. The EVs were modified with a CDH7-specific nanobody (E8) and loaded with croconaine, a NIR dye (CR@E8-EVs). This exhibited enhanced stability, efficient clearance, minimal off-target effects, active tumor targeting, and significant retention, enabling precise photoacoustic imaging-guided PTT for gastric cancer. (B) Optical imaging and biodistribution of E8-EVs in tumor-bearing mice. (C) Photothermal imaging, tumor growth, body weight, and histological analysis showing the therapeutic efficacy. TUNEL and Ki67-positive staining were performed to confirm the cell quantification. The data also included whole-body, *ex vivo* imaging, and histology across treatment groups, with relevant scale bars indicated^156^. Reprinted with permission Ref. 156. Copyright © 2023, Springer Nature.

Fluorescence imaging explores EV distribution within a biological system. For monitoring, EVs are labeled with lipophilic carbocyanine dyes like PKH67, PKH26, DiI, MemGlow-488 (MG-488), or DiR^121^, ^122^, ^123^^,^^125^. This fluorescent labeling offers insights into clinical translation by evaluating the effectiveness of EV-mediated treatment in preclinical cancer models. However, the prolonged presence of the fluorescent dyes in tissues and nonspecific lipid labeling leads to inaccurate tissue identification^119^^,^^123^^,^^126^. Some commonly used fluorescent proteins include GFP, MyrPalm-superFolderGFP (mp-sfGFP), and RFP^125^^,^^127^^,^^128^. Fluorescent proteins production requires transfection of producer cells with a plasmid encoding the protein, followed by isolation of the labeled EVs^129^. Studies have demonstrated that the role of EVs expressing CD63-GFP increases targeted secretion of miRNAs in mice^130^. Additionally, croconaine-loaded E8–engineered EVs (CR@E8–EVs) showed strong NIR absorption, tumor-specific targeting, and effective photothermal ablation upon laser irradiation. This biocompatible platform enables precise imaging and potent tumor treatment, offering a promising approach for cancer theranostics^131^ (Fig. 6). Metabolic labeling of small EVs (sEVs) with Bodipy FL C16 is also demonstrated for cancer treatment. Fluorescent palmitic acid incorporated into a specific subpopulation of sEVs improves their visualization and tracking through the distinct fluorescence signal^132^. In summary, incorporating fluorescent dyes or probes into EVs can offer key insights into their biodistribution, uptake, and therapeutic effectiveness, helping to speed up their development as cancer treatments.

#### 6.1.1 Quantum dot labeling

**Figure 7:**
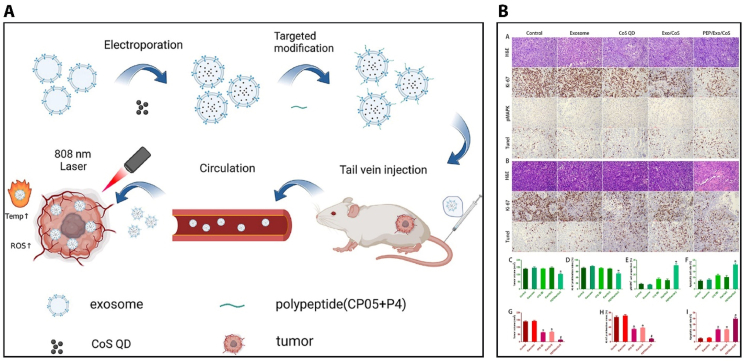
Targeted delivery and synergistic therapy of peptide/exosome/cobalt sulfide quantum dots (PEP/Exo/CoS QDs) in bladder cancer treatment. By combining the high specificity of the platform with the combined effects of chemodynamic and photothermal therapies. (A) A schematic illustration showing the mechanism involved in the targeted delivery and the synergistic action of chemodynamic and photothermal therapies in bladder cancer treatment. (B) Ki-67 proliferation index, pMAPK expression, and apoptosis were assessed by using immunohistochemistry and TUNEL staining in the unirradiated group compared to the irradiated group. The results demonstrated the significant anticancer effects of PEP/Exo/CoS QDs *in vivo*, highlighting their potential in tumor treatment^172^. Reprinted with permission from Ref. 172. Copyright © 2023, Elsevier.

Quantum dots (QDs) are a promising approach that involves the use of semiconductor nanocrystals, which have distinct optical and electronic properties^133^^,^^134^. QDs are suitable for multiple imaging techniques, such as fluorescence imaging, photoacoustic imaging, and computed tomography^135^. For instance, QDs have been exploited for both therapy and imaging due to their bright fluorescence, high photostability, and tunable emission spectra^135^. Additionally, QDs can be conjugated with chemotherapeutic drugs, photosensitizers for photodynamic therapy, or RNA molecules for gene therapy. This enables targeted delivery and controlled release of therapeutic payloads, resulting in enhanced treatment effectiveness with reduced side effects^106^^,^^136^, ^137^, ^138^, ^139^, ^140^, ^141^, ^142^, ^143^, ^144^. Studies have also shown that the natural lipid bilayer of EVs offers a biocompatible and nonimmunogenic platform for QD delivery, increasing their pharmacokinetics, biodistribution, and tumor targeting ability^106^^,^^138^, ^139^, ^140^, ^141^, ^142^, ^143^, ^144^. For example, Zhang et al.^145^ showed that the chiral graphene QD-sEV platform effectively delivers drugs to tumor cells, reducing tumor growth with good biocompatibility and fewer side effects. A dual-targeting peptide/exosome/cobalt sulfide QD platform in tumor tissues produces significant antitumor effects *via* photothermal and chemodynamic mechanisms in bladder cancer mouse model. This approach minimizes damage to normal tissues during laser irradiation (Fig. 7)^146^.

In conclusion, addressing the challenges faced during QD-based EVs for imaging-guided therapy requires improved loading efficiency and stability of QDs in EVs. These improvements reduce QD toxicity and immunogenicity, and pave the way for standardized large-scale production and quality control of EV-QD formulations.

### 6.2 Bioluminescence labeling

**Figure 8:**
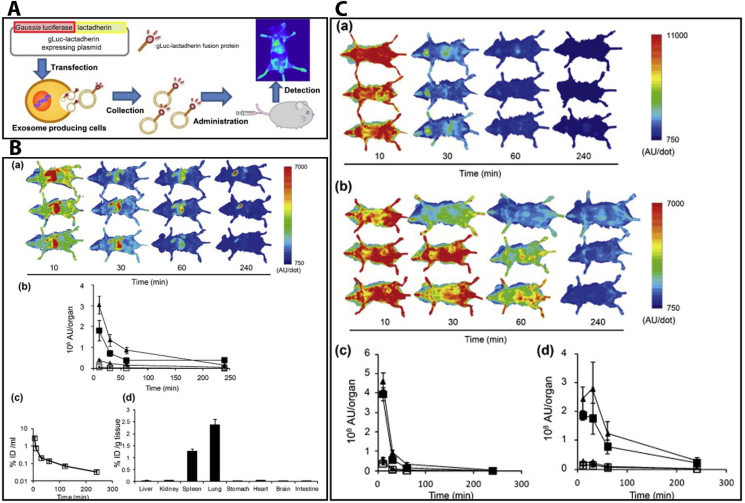
GLuc-Lactadherin labeling for *in vivo* tracking of B16-BL6 Exosomes. (A) A schematic illustration showing GLuc-cadherin labeling for tracking B16-BL6 exosomes in human liver and lung tissues. The rapid clearance of these exosomes from circulation after systemic administration emphasizes the potential of GLuc-lactadherin labeling in studying exosome biodistribution and clearance mechanisms, aiding the development of improved therapeutic and diagnostic strategies. (B, C) BALB/c mice were intravenously injected with exosomes derived from pCMV-gLuc-lactadherin-transfected B16BL6 cells. The gLuc-expressing B16-BL6 exosomes were imaged at various time points following a bolus intravenous injection of coelenterazine. The time course of chemiluminescence intensity was tracked in different organs, as well as the gLuc activity in serum after injection of the GL-labeled exosomes in C57BL/6 mice. Tissue distribution of gLuc activity was assessed 4 h post-injection. Additionally, BALB/c mice received intravenous injections of exosome-free gLuc protein or gLuc-lactadherin protein, and gLuc activity was assessed over time, with chemiluminescence intensity recorded in various organs after protein injection^175^. Reprinted with permission from Ref. 175. Copyright © 2013, Elsevier.

Bioluminescence is a tool for visualizing dynamic biological processes due to its high signal-to-noise ratio, ability to image multiple organisms at once, improved sensitivity and specificity, lack of phototoxicity, and non-invasive characteristics^147^. In contrast to fluorescence labeling, bioluminescence relies on light emission resulting from a natural enzyme-substrate reaction rather than requiring excitation and emission^129^. Bioluminescent labeling of EVs includes luciferase enzymes like firefly luciferase (FLuc), ThermoLuc, NanoLuc, *Renilla* luciferase (RLuc), and Gaussian luciferase (GLuc)^148^. For example, NanoLuc and ThermoLuc enable sensitive *in vivo* biodistribution assessment and robust EV tracking^148^. Studies have shown that GLuc and lactadherin labeling allow profiling of B16–BL6 exosomes in the human liver and lungs. This highlights the use of GLuc-cadherin labeling to track exosome biodistribution and clearance with rapid elimination from circulation following systemic administration (Fig. 8)^149^. RLuc is also employed for EV monitoring within live cells in *in vivo* bioluminescence imaging, and the combination of GLuc with biotinylation can result in the development of an EV reporter for multimodal *in vivo* imaging^150^^,^^151^. The limitation of bioluminescence labeling is the difficulty in penetrating deep tissues due to limited light emission^152^. Additionally, the need for genetic modification in parent cells is challenging, particularly for exosomes derived from physiological fluids like blood or urine, where genetic modification is often unfeasible^153^. For example, primary cells show lower transfection efficiency compared to immortalized cell lines^149^. In conclusion, bioluminescence labeling is a promising technique for non-invasive visualization of engineered EVs *in vitro* and *in vivo*.

### 6.3 Radioactive isotope labeling

Radiolabeling is a technique that uses radioactive isotopes to track isotopic movement during reactions, metabolic processes, or within cells^141^^,^^154^. Exosomes labeled with clinical-grade radioactive isotopes like ^99m^Tc, I-131, and ^111^In-oxine enable reliable *in vivo* distribution tracking. This approach provides both qualitative and quantitative assessments, even with some degradation of the labeled exosomes, and allows imaging of deeper tissues due to the isotopes’ tissue-penetrating properties^155^. EVs are typically labeled with indium and ^99m^Tc-HMPAO, as the lipophilic ^99m^Tc-HMPAO interacts with reducing agents in EVs, which converts them to a hydrophilic form. Meanwhile, ^111^In is more affordable and provides superior image resolution for gamma camera imaging compared to ^99m^Tc-HMPAO^156^. Hwang et al.^157^ showed that ^99m^Tc-HMPAO-labeled macrophage-derived exosome-mimetic nanovesicles (ENVs) retained high radiochemical purity and stability in serum, with preferential uptake in the liver. Molavipordanjani et al.^158^ also showed that radiolabeling genetically modified tumor-derived EVs with ^99m^Tc led to high accumulation in HER2-expressing tumors in SKOV-3 tumor-bearing nude mice. These findings confirmed the effectiveness of ^99m^Tc-radiolabeled exosomes for targeted tumor imaging and therapy.

**Figure 9:**
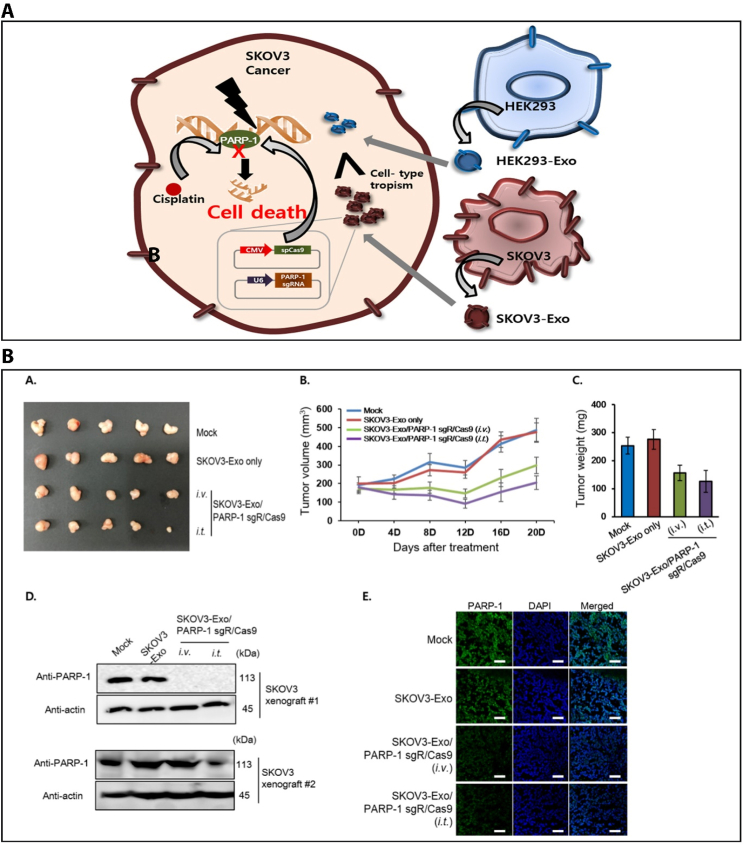
Cancer exosome-based combination therapy against ovarian cancer. (A, B) Illustrating the effective targeting of PARP1 by using SKOV3-derived exosomes loaded with CRISPR/Cas9 in combination with cisplatin to suppress cancer^136^. Reprinted with permission from Ref. 136. Copyright © 2017, Elsevier.

Radiolabeling is a biomedical tool to assess exosome behavior, as shown in Fig. 9. This is achieved by modifying the EV surface with a radioactive substance through chemical ligation. For instance, EVs labeled with ^111^In in combination with intraluminal, incubation, and membrane techniques showed improved labeling efficiency and radiochemical stability compared to intraluminal labeling alone in melanoma cells^159^. ^111^In is often favored for imaging due to its longer half-life compared to other radioiodine isotopes (^123^I, ^124^I, ^125^I, ^131^I) that have unique physical characteristics^159^^,^^160^. Live monitoring of radiolabeled exosomes is feasible through non-invasive approaches such as single-photon emission computed tomography (SPECT) or positron emission tomography (PET)^155^. Studies have demonstrated the used of SPECT/computed tomography (CT) to understand the drug kinetics and tissue distribution of exosomes, underscoring the effectiveness in drug delivery^161^. Furthermore, anatomical imaging is used for CT or magnetic resonance imaging (MRI). MRI has been utilized to track superparamagnetic iron oxide nanoparticle (SPION)-loaded exosomes traveling to lymph nodes, offering insights into exosome biology and their impact on lymph node architecture in melanoma^162^. Additionally, *ex vivo* examination of organs is commonly used for imaging or studying the biodistribution of exosomes.

### 6.4 Magnetic nanoparticle labeling

**Figure 10:**
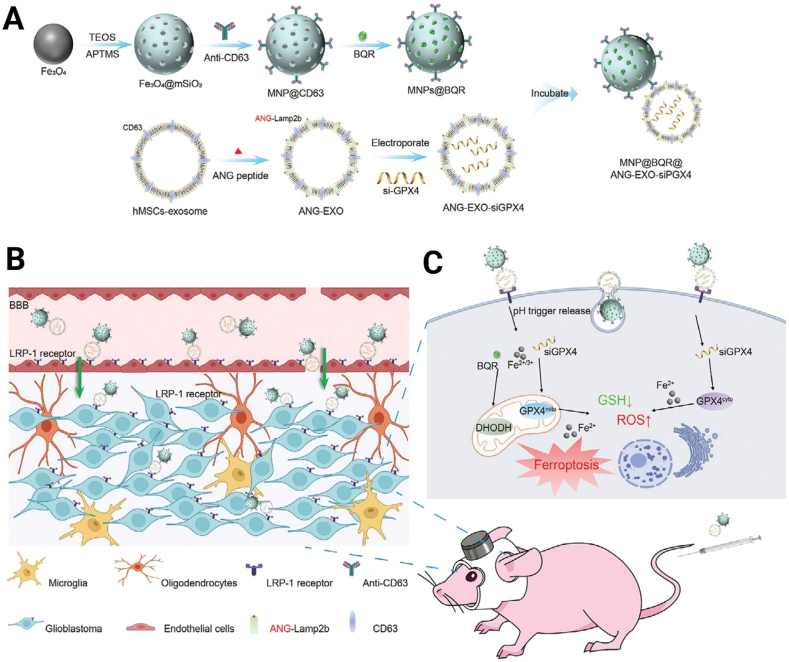
Design, synthesis, and mechanism of MNP@BQR@ANG-EXO-siGPX4 for targeted glioblastoma (GBM) therapy. A) Schematic depiction of the design and synthesis process for MNP@BQR@ANG-EXO-siGPX4. B) Illustration of a magnetic mouse helmet and the process by which ANG peptide-modified nanoparticles traverse the blood-brain barrier (BBB) to target tumors. C) Mechanism of ferroptosis induction in GBM cells^226^. Reprinted with permission from Ref. 226. Copyright © 2022, Advanced Science published by Wiley.

**Figure 11:**
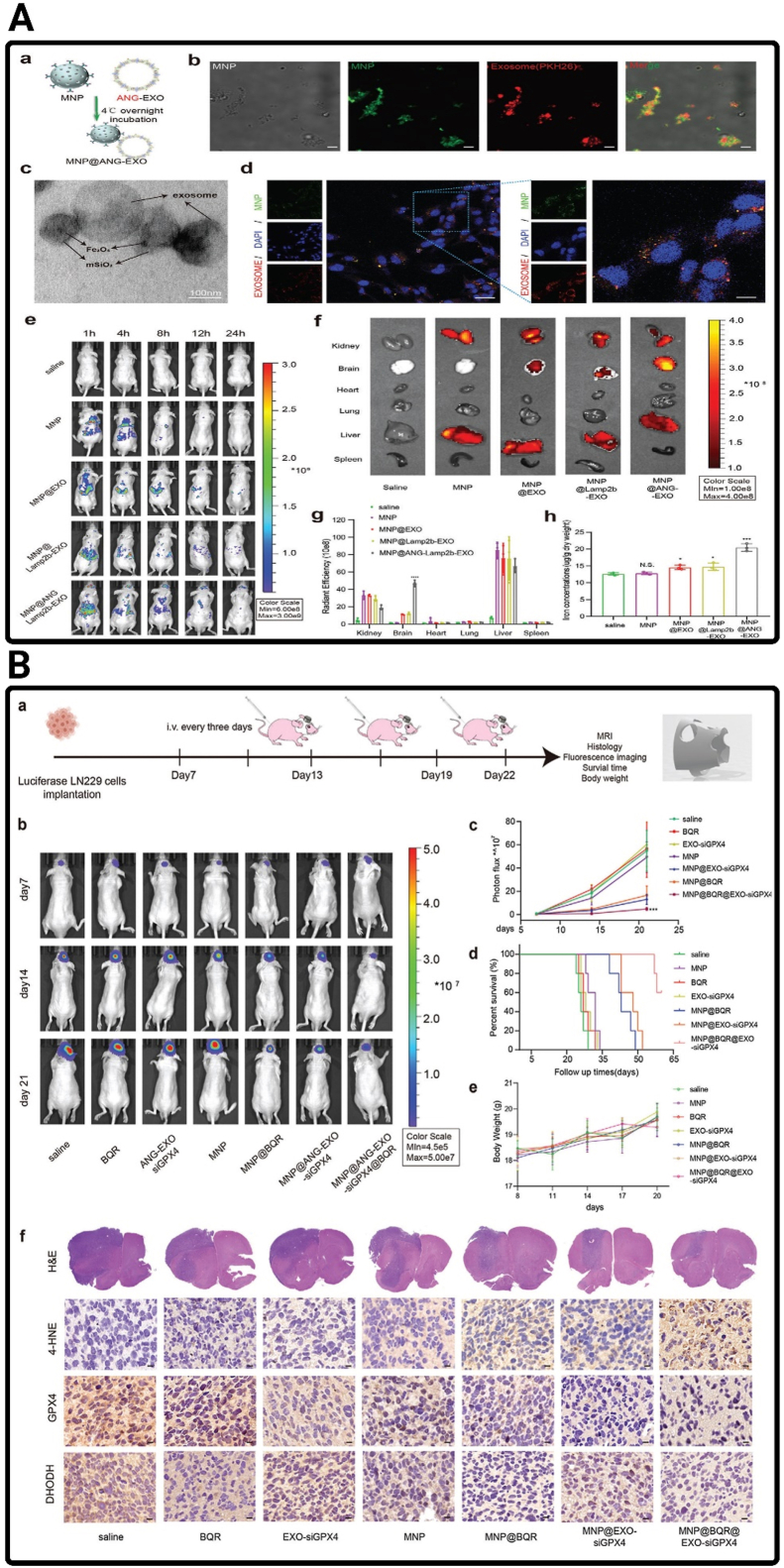
Targeting ability of MNPs@ANG-EXO *in vitro* and *in vivo*. (A) a) Schematic showing MNPs bound to exosomes following incubation at 4 °C. b) Confocal microscopy images showing the colocalization of exosomes (red, PKH26) with magnetic NPs (green, FITC). Scale bar, 5 μm. c) TEM image of exosome-conjugated magnetic nanoparticles. Scale bar, 100 nm. d) Cellular uptake of MNP@ANG-EXO in LN229 cells after 6 h. Scale bar, 50 μm (left), 10 μm (right). e) *In vivo* distribution of saline, MNPs, MNP@EXO, MNP@Lamp2b-EXO, and MNP@ANG-EXO in orthotopic DIPG-bearing mice 24 h postinjection. f, g) *Ex vivo* fluorescence images of organs after magnetic field-guided injection and fluorescence quantitative analysis (*n* = 3; ∗∗∗∗*P* < 0.0001 *vs*. saline). h) ICP–MS detection of brain iron levels (*n* = 3; ∗*P* < 0.05, ∗∗∗*P* < 0.001 *vs*. saline). Data: mean ± SD. (B) Antitumor efficacy of MNP@BQR@ANG-EXO-siGPX4. a) Timeline and 3D-printed mouse helmet model in mouse. b) Luminescence images of orthotopic LN229-Luc + GBM tumors after treatments on Days 7, 14, and 21. c) Luminescence quantitative analysis (*n* = 5; ∗∗∗ *P* < 0.001 *vs*. saline). d) Survival curves of mice (*n* = 5). e) Mouse body weight changes during treatment (*n* = 5). f) H&E staining and immunohistochemistry of 4-HNE, GPX4, and DHODH in GBM tumors post-treatment. Scale bar, 10 μm. Data: mean ± SD^226^. Reprinted with permission from Ref. 226. Copyright © 2022, Advanced Science published by Wiley.

Magnetic nanoparticles (MNPs), typically made of iron oxide (Fe_3_O_4_ or *γ*-Fe_2_O_3_), possess superparamagnetic properties, allowing them to respond to magnetic fields without permanent magnetization. Their small size and surface modification capabilities make them suitable for biomedical uses such as MRI, drug delivery, cell separation, and hyperthermia therapy^163^, ^164^, ^165^. Guo et al.^166^ developed DOX-loaded exosome mimetics that enabled precise magnetic targeting and effective tumor suppression in breast cancer. Qi et al.^167^ also used blood-derived exosomes combined with superparamagnetic nanoparticles to enhance drug delivery to tumors with minimized off-target effects. In a preclinical study, magnetic nanoparticles and engineered exosomes induce ferroptosis by targeting DHODH and GPX4, further releasing Fe^2+^ ions in glioblastoma. Angiopep-2-modified exosomes crossed the blood–brain barrier and target GBM cells *via* LRP-1 receptors, resulting in improved tumor inhibition and prolonged survival (Figure 10, Figure 11). MNPs have also revolutionized exosome isolation by offering rapid and efficient techniques^168^. Additionally, Huang et al.^168^ developed magnetic graphene oxide nanoparticles with CD63 aptamers, resulting in 89.4% exosome capture in 15 min while maintaining structural integrity. Likewise, Lim et al.^169^ used antibody-linked magnetic nanowires to achieve higher yield and purity, improving biomarker detection for cancer diagnosis. Meanwhile, for *in vivo* imaging like MRI-based visualization, SPIONs have been widely used to label exosomes^162^. Studies indicate that melanoma exosomes labeled with SPIONs improve lymph node targeting capabilities through MRI imaging^162^. Additionally, Liu et al.^170^ demonstrated that the introduction of ferritin heavy chain (FTH1) as an endogenous MRI reporter to track exosome dynamics *in vivo* offers a non-invasive approach to study cancer progression. Magnetic hyperthermia therapy employs MNPs and alternating magnetic fields to generate heat for cancer cell destruction. Altanerova et al.^171^ used iron oxide-labeled MSC-derived exosomes to enhance tumor ablation and combined this with gene therapy for dual therapeutic benefits.

MNPs are also valuable in exosome-based diagnostics. Zhu et al.^172^ (2021) developed a magnetic aptamer sensor with FRET for ultra-sensitive lung cancer exosome detection, achieving 100% accuracy. Li et al.^173^ also developed a magnetic SERS platform to isolate and analyze breast cancer exosomes with high sensitivity and specificity. Additionally, MNPs enhance tumor tracking and biodistribution analysis *in vivo*. In addition, Toomajian et al.^175^ also highlighted that SPIO-labeled EVs can cross the BBB and track brain metastases. Zhuang et al.^176^ demonstrated the effectiveness of SPION-decorated exosomes loaded with TNF-*α*, resulting in targeted cancer therapy under magnetic guidance. Furthermore, Wei et al.^177^ showed that the introduction of bacteria-responsive magnetic exosomes is safe and accurate for tumor imaging.

## 7 Engineered EVs for alternative cancer combination therapy approaches

**Figure 12:**
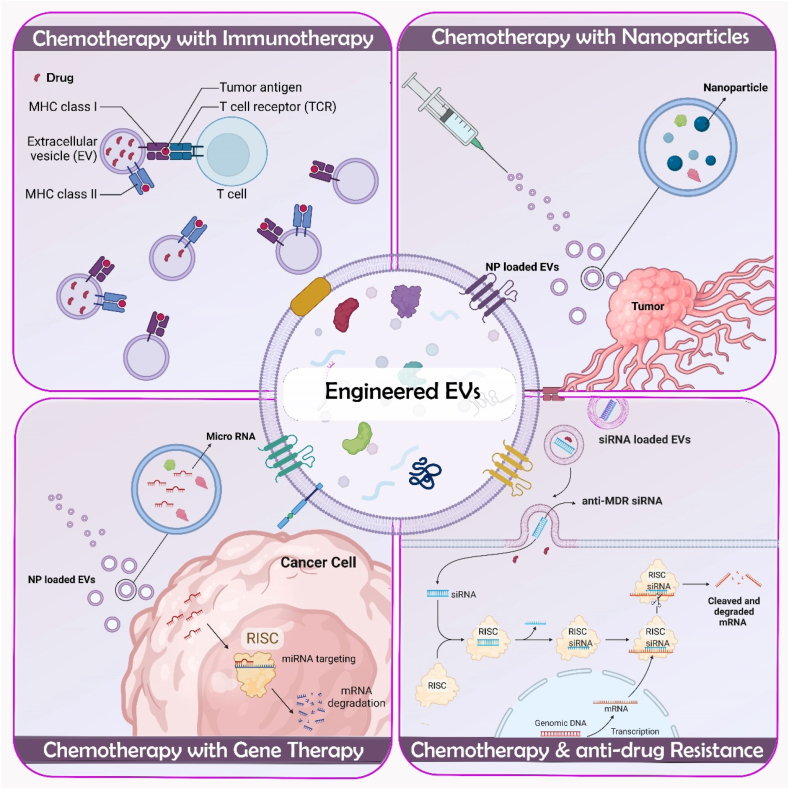
Overview of engineered EVs in combination cancer therapies. This chart highlights strategies that integrate EV-based delivery like radiotherapy, photodynamic, photothermal, and sonodynamic therapies. This harness the unique EVs features to enhance treatment effectiveness and precision with reduced side effects in cancer treatment.

Several studies have explored how engineered EVs could improve combination therapies (Fig. 12). Studies have shown the self-activation of photoEVs from M1 macrophages in combination of immunotherapy, PDT, and chemotherapy to enhance anticancer effects. These EVs carry bis[2,4,5-trichloro-6-(pentyloxycarbonyl) phenyl]oxalate (CPPO), the photosensitizer Ce6, and the prodrug aldoxorubicin (Dox-EMCH). The reaction between H_2_O_2_ and CPPO generates energy that activates Ce6 to produce singlet oxygen for PDT then disrupts the EV membrane to release Dox-EMCH for chemotherapy. This trimodal therapy showed effectiveness in mice with 4T1 tumors^178^. Another study demonstrated a multimodal therapy using exosomes loaded with DOX, encapsulated in magnetic nanoparticles, and attached to molecular beacons for imaging. This precision nanomedicine platform uses magnetic nanoparticles for targeted tumor delivery guided by an external magnetic field, which also triggers localized hyperthermia *via* NIR radiation. Combining chemotherapy, gene therapy, and PTT under magnetic guidance led to high cancer cell killing (91.04%) and a significant tumor reduction (97.57%)^179^**.**

Sonodynamic therapy (SDT) uses ROS generated by sonosensitizers and ultrasound to target tumors with increased effectiveness when combined with other therapies, as compared to SDT alone^180^. For example, encapsulating ICG, paclitaxel (PTX), and sulfadiazine (SBC) in exosomes serves as dual stimuli-responsive sonosensitizers. This approach enables pH-sensitive photoacoustic imaging to guide the combined use of chemotherapy and SDT, improving cancer treatment^180^. Furthermore, combining PTT with hyperthermia induced by photothermal agents (PTAs) and NIR laser light can facilitate drug release and effectively kill tumor cells^181^^,^^182^.

Incorporating ICG into exosomes with high CD47 expression, along with a sonosensitizer and photoacoustic contrast agent, enhances ICG-mediated PTT^180^^,^^183^^,^^184^. Furthermore, an exosome-based system also used camouflaged porous silicon NPs to co-deliver ICG and DOX, while polydopamine (PDA) on the exosome surface acts as a photothermal agent, converting NIR light into heat^185^^,^^186^. A study has showed that this heat triggers rapid release of DOX and miR-21 resulting in a multimodal therapeutic effect^187^.

## 8 Engineered EVs for combination therapies related to chemotherapy

Chemotherapy is a key cancer treatment, but faces challenges including systemic delivery, multidrug resistance (MDR), and tumor cell heterogeneity^188^^,^^189^. The use of EV-mediated carrier systems to deliver chemotherapy drugs has improved the effectiveness of the therapy. Aptamer-engineered exosomes can precisely target and deliver DOX into colorectal cancer cells^190^. The development of engineered EVs has led to new strategies to increase tumor-killing efficacy with reduced chemotherapy side effects^191^. These advanced EVs are customized for various chemotherapy combinations with other therapies, such as drug resistance treatments, nanoparticle therapy, gene therapy, and immunotherapy (Table 4)^192^.

**Table 4:** Potential cancer therapies using engineered EVs.

Scheme	Principle/Mechanism	EV Origin	EV type	Modified strategy	Cargoes	Membrane modification	Outcome	Ref.
Chemotherapy combined with therapies to overcome resistance	Codelivery of P-gp siRNA and DOX by modified EVs	RBC	Mimic vesicles	Incubation	P-gp siRNA and DOX	Aptamer engineering	Overcome resistance and target MDR- tumors	273
	Tumor cell-derived EVs reduce P-gp expression	Bel7402 cells	Biomimetic EVs	Incubation	PSINPs loaded with DOX	N/A	Cellular uptake and cytotoxicity in cancer cells	274
		HEK293T cells	EVs	Incubation	DOX	LipHA engineering	Reduced MDR tumor growth and increased survival	275
	Codelivery of anti-miRNA and drugs	HCT-1165FR cell	Exosomes	Electropermeabilization	MiR21i and 5-FU	N/A	Address drug resistance and enhance cytotoxicity in colon cancer cells	143
		HEK293T cell	Exosomes	Transfection	Anti-miR-214	N/A	Countered resistance to CDDP in gastric cancer	276
		4T1 cells	Tumor cell-derived EVs	EVs from cells transfected with anti-miR-21	Anti-miR-214 and GIONs	N/A	Decreased resistance, improved photothermal effectiveness and enhanced T2 MR imaging capabilities	276
Combination of chemotherapy with PTT	Dual delivery of PTA and chemotherapy	DC2.4	EVs	Incubation	DOX	Gold nanoparticles	Controlled drug release and minimized adverse effects	277
		H22 cells	Microparticles	Electropermeabilization	Bi2Se3 nanodots combined with DOX	N/A	Improved antitumor efficacy	278
		HeLa cells	Microvesicles	Electropermeabilization	ICG and DOX	N/A	High anticancer activity through the release of DOX and ICG	279
		4T1 cells	Biomimetic exosomes	Exosomes combined with MSNs and processed through extrusion	ICG and DOX	N/A	High effectiveness to chemo-PTT	280
Combination of chemotherapy with gene therapy	Exosomes delivery of CRISPR/Cas9	SKOV3 cells	Cancer-derived exosomes	Electropermeabilization	N/A	Targeting PARP1 with CRISPR/Cas9	Increased cancer cell apoptosis and enhanced CDDP chemosensitivity	169
	Dual delivery nucleic acids therapy and chemotherapy	Cal 27 cells	Microvesicles (MVs) were from modified parent cells	Bcl-2 siRNA and PTX infected into MVs by electropermeabilization	Bcl-2 siRNA and PTX	Biotin and folate	Precise targeting of breast cancer with high efficacy	281
Dual delivery of nanoparticles and drugs	Modified chemotherapy loaded with NPs	MDA-MB-231 cells	Biomimetic exosomes	Coextrusion of PCNPs and exosome membrane	PTX-S-LA and CuB loaded with PEG-PCL NPs	N/A	Impede breast cancer metastasis	282
		Macrophages	Biomimetic exosomes	Coextrusion of exosomes mixed with PLGA	Poly lactic-co-glycolic acid (PLGA) nanoparticles loaded with DOX	N/A	Precise tumor-targeting reduced tumor growth	283
Combination of immunotherapy with chemotherapy	Dual delivery of chemotherapy and immunomodulator	Fibroblasts	Thermosensitive exosome--liposome hybrid NPs	Integration of modified exosomes with drug-loaded thermosensitive liposomes	GM-CSF and docetaxel	High levels of CD47	Increased CD47 expression	284
		BM-MSCs	Exosomes	Galectin-9 siRNA with OXA-MAL loaded into exosomes	Galectin-9 siRNA	OXA	High anti-tumor activity by suppressing tumor growth	285
Combination of immunotherapy with PDT or PTT	Dual delivery of immunostimulatory and PS or PTA while PDT and PTT induce ICD and covert “cold” tumor into “hot” tumor	MSCs	EVs	Turbulence-driven generation of MSC-derived EVs encapsulating mTHPC	Photosensitizer mTHPC	N/A	High tumoral necrosis	286
		CT26 cells	Hybrid vesicles	Integration of exosomes and thermosensitive liposomes	ICG and adjuvant R837	High level of CD47	Reduced tumor growth	264
Combination of ICI with anti-ICI resistance therapy	Anti-ICI resistance therapy improved ICI resistance	RAW264.7 macrophages	Microparticles	RAW264.7 macrophages incubated with DSPE-PEG-Man and then treated with metformin	Metformin	Mannose	High anticancer efficacy	287
Combination of cancer vaccine with immune checkpoint blockade	Modified EVs as vaccines and ICI increased vaccine efficacy	B16F10 cells/CT26 cells	Tumor-derived microparticles (T-MPs)	MPs containing CpG-loaded liposome while nano-Fe3O4 incubated with parental cells	Nano-Fe3O4	CpG-loaded liposome	Impede tumor growth	288
		BMDCs	Exosomes	Ovalbumin-pulsed exosomes were modified with anti-CTLA-4 antibody and activated DCs	N/A	Anti-CTLA-4 antibody	Reduced tumor progression	289
Hybrid image-guided PTT	Modified goat milk-derived EV (GMVs)	N/A	EVs derived from goat milk	PTT nanocarriers	N/A	Nanoprobe employed for PET/CT/NIRF imaging and image-guided PTT	High anti-tumoral immunity due to increased PTT in colon cancer	290
Reprogramming of T-cell-derived small EVs	IL-2-tethered sEVs resident microRNAs	Jurkat T cells	EVs	Modified Jurkat T cells with IL2 expressed on their plasma membrane *via* a flexible linker to trigger an autocrine response	miR-181a-3p and miR-223-3p	N/A	High CD8+ T-cell potency with reduced cellular and exosomal PD-L1 expression in melanoma cells	291
Modified EVs for anti-PDL1 immunotherapy and chemotherapy	N/A	MDA-MB-231	EVs	High-affinity variant human PD-1 protein (havPD-1)	N/A	N/A	High anti-tumor activity *via* PD-L1 blockage leads to T-cell suppression	292

EVs, Extracellular vesicles; P-gp, P-glycoprotein; DOX, doxorubicin; RBC, red blood cell; MDR, multidrug resistance; PSINPs, porous silicon nanoparticles; LipHA, lipid-grafted hyaluronic acid; 5-FU, 5-fluorouracil; GIONs, gold–iron oxide nanoparticles; PTA, photothermal agents; PTT, photothermal therapy; ICG, indocyanine green; E-MSNs, exosome-camouflaged mesoporous silica nanoparticles; PARP1, poly (ADP‒ribose) polymerase-1; MV, microvesicles, NP, nanoparticles; PLGA, poly (lactic‒iron glycolic acid); GM-CSF, granulocyte‒macrophage colony-stimulating factor; OXA, oxaliplatin; MAL, N-(2-Aminoethyl) maleimide; PDT, photodynamic therapy; PTT, photothermal therapy; PS, photosensitizers; PTA, photothermal agents; ICD, immunogenic cell death; ICG, indocyanine green; mTHPC, meta(tetrahydroxyphenyl)-chlorin; ICI, immune checkpoint inhibitors.

### 8.1 Chemotherapy combined with anti-drug resistance treatment based on engineered EVs

MDR accounts for approximately 90% of chemotherapy failures, which is attributed primarily to inherent or acquired mechanisms in cancer cells^193^. These mechanisms include increased activity of drug efflux pumps, heightened DNA damage repair, dysfunction in apoptosis, epigenetic alterations, and changes in drug metabolism and cellular targets^194^. Several approaches to overcome MDR include using efflux pump inhibitors alongside anticancer drugs. This could be carried out by using RNA interference to silence MDR-related genes and developing drugs that can counteract, avoid, or take advantage of efflux mechanisms^195^. Engineered EVs present a promising avenue to reverse MDR in tumors by selectively accumulating anti-MDR substances and chemotherapeutic drugs at tumor sites through passive or active targeting, thereby enhancing chemotherapeutic effects^192^. These findings indicate that tumor cell-derived EVs directly reverse drug resistance by reducing the expression of P-glycoprotein (P-gp)^196^. For example, the use of CDDP-loaded tumor cell-derived microparticles reduces drug resistance by blocking drug efflux pumps, increasing the nuclear import of drugs, and interacting with lysosomes *via* unique membrane structures enriched with lipid rafts^197^.

**Figure 13:**
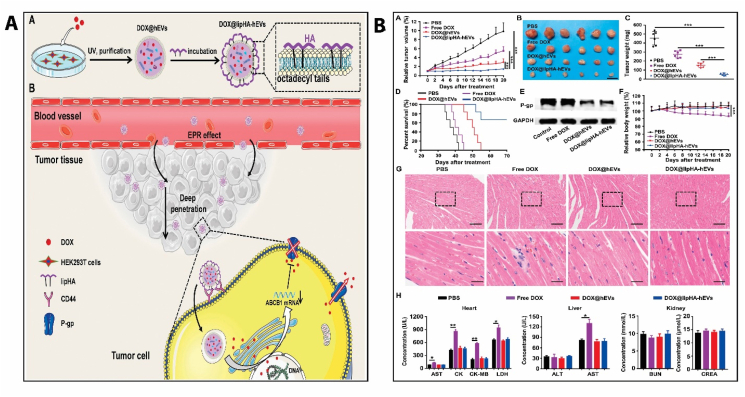
Schematic representation of drug-resistant tumor therapy using hyaluronic acid (HA)-functionalized EVs (lipHA-hEVs). (A) This panel shows how lipHA-hEVs are prepared by modifying EVs with lipid-grafted hyaluronic acid to enhance their effectiveness against drug-resistant tumors. It also illustrates their targeted delivery process and key therapeutic actions, including precise drug delivery and the suppression of drug efflux. (B) This study compares treatments in mice over 21 days, showing that lipHA-hEVs outperform PBS, free DOX, and DOX@hEVs in reducing tumor size and weight. Tumor images, survival analysis, and decreased P-gp levels highlight the effectiveness of lipHA-hEVs in inhibiting drug efflux and improving survival. Body weight monitoring and heart tissue staining indicate good overall health, while serum tests confirm the safety and tolerability of the treatment^233^. Reprinted with permission. From Ref. 233. Copyright © 2019, Elsevier.

One critical MDR strategy involves high levels of efflux transporter pump receptors, such as P-gp, in the cell membrane^197^. P-gp, known as multidrug resistance protein 1 (MDR1), pumps anticancer substances out of cells, preventing their accumulation within the cell^196^. Co-delivery of P-gp-targeted siRNA (MDR-1 siRNA) and chemotherapeutic drugs through engineered EVs effectively inhibits drug-resistant tumors. For example, aptamer-modified mimic vesicles designed for targeted siRNA delivery and DOX demonstrated synergistic activity in overcoming drug resistance^198^. Another study showed that engineered EVs modified with hyaluronic acid (lipHA-hEVs) effectively delivered chemotherapy drugs by targeting CD44. These vesicles reduced drug efflux by decreasing P-gp levels and enhanced treatment by colocalizing with lysosomes^199^. In a preclinical MDR tumor model, lipHA-hEVs efficiently delivered DOX to tumors, minimizing systemic toxicity and suppressing tumor growth by 89% (Fig. 13)^199^. Similarly, DOX-loaded porous silicon nanoparticles coated with exosomes and modified with hyaluronic acid improved drug accumulation in tumors and lowered P-gp levels, altering cell membrane fluidity. These findings highlight the promise of engineered EVs in reducing P-gp expression and combating multidrug resistance^199^^,^^200^.

Elevated ROS levels and the regulation of redox balance by antioxidants like glutathione (GSH) contribute to the development of MDR in cancer cells. For example, Kang et al. designed GSH-depleting benzoyloxy dibenzyl carbonate (B2C)-encapsulated small EVs (BsEVs) to trigger oxidative stress and cell death in ovarian cancer cells. These BsEVs effectively counteract MDR by disrupting mitochondrial function and ATP synthesis, thereby impairing efflux pumps and enhancing drug responsiveness in resistant ovarian cancer cells.

miRNAs, such as miR-22, miR-214, miR-19, miRNA-17-5, and miR-221, post-transcriptional regulatedrug-resistance genes across various cancers^202^^,^^203^. Modulating miRNAs *via* competitive inhibitors such as ASOs is an important strategy for combating drug resistance^204^. Studies have explored engineered EVs to improve chemotherapy efficacy by combining miRNA inhibitors, such as anti-miR-214 and anti-miR-21, with chemotherapeutic agents such as CDDP and DOX in cancer cells^205^. For example, administering 5-fluorouracil (5-FU) and miR-21 inhibitors with surface-engineered exosomes bearing Her2 and LAMP2 significantly reduced tumor growth and drug resistance in a colon cancer mouse model^206^. These findings highlight the effectiveness of modified exosomes in addressing drug resistance and improving chemotherapy efficacy by increasing the therapeutic agent in the TME.

### 8.2 Modified EVs for the combination of chemotherapy and gene therapy

Gene therapy has promising effects on cancer treatment by targeting genetic mutations that drive cancer and affect treatment outcomes through the replacement of defective genes with functional genes^207^^,^^208^. This approach can increase antitumor drug efficacy and minimize side effects^209^. Strategies such as suicide genes, ribozymes, and nucleic acid-based drugs such as antisense oligonucleotides and RNAi are being explored to counteract malignant transformation and fight cancer^210^, ^211^, ^212^.

**Figure 14:**
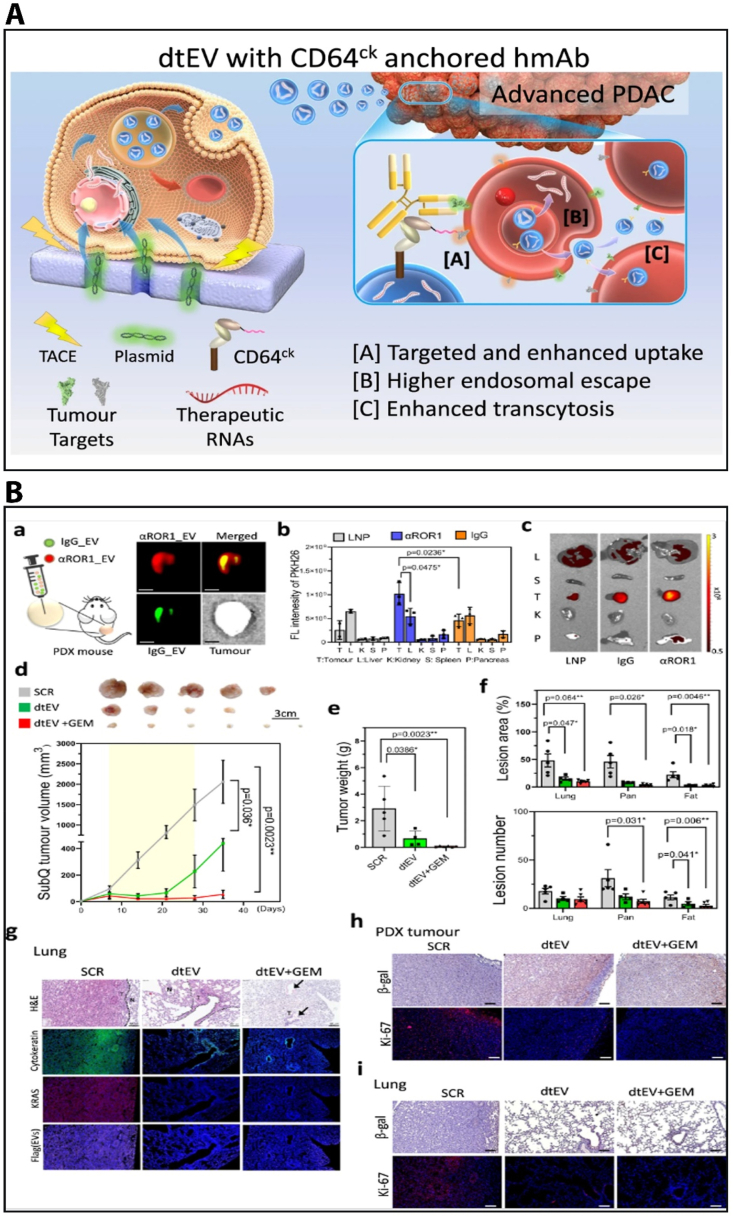
Overview mechanism for dual-targeted EVs (dtEVs) regulating oncogenic genes in advanced pancreatic cancer. (A) A schematic for dual-targeted EVs containing therapeutic RNA and the CD64 (Fc-gamma receptor 1) protein engineered at the N-terminus with a CKAAKNK (CK) tissue-homing peptide that targets pancreatic tumor tissue (CD64CK protein) for cancer-specific delivery of CRISPR/Cas9. (B) In a patient-derived xenograft mouse model, dtEVs showed strong tumor penetration and specific tumor accumulation. Treatment with dtEVs, especially when combined with GEM, significantly reduced tumor size, weight, and metastases. Tissue staining and biomarker analysis confirmed decreased tumor cell proliferation and metastatic spread^249^. Reprinted with permission from Ref. 249. Copyright © 2023, Nature Communications.

**Figure 15:**
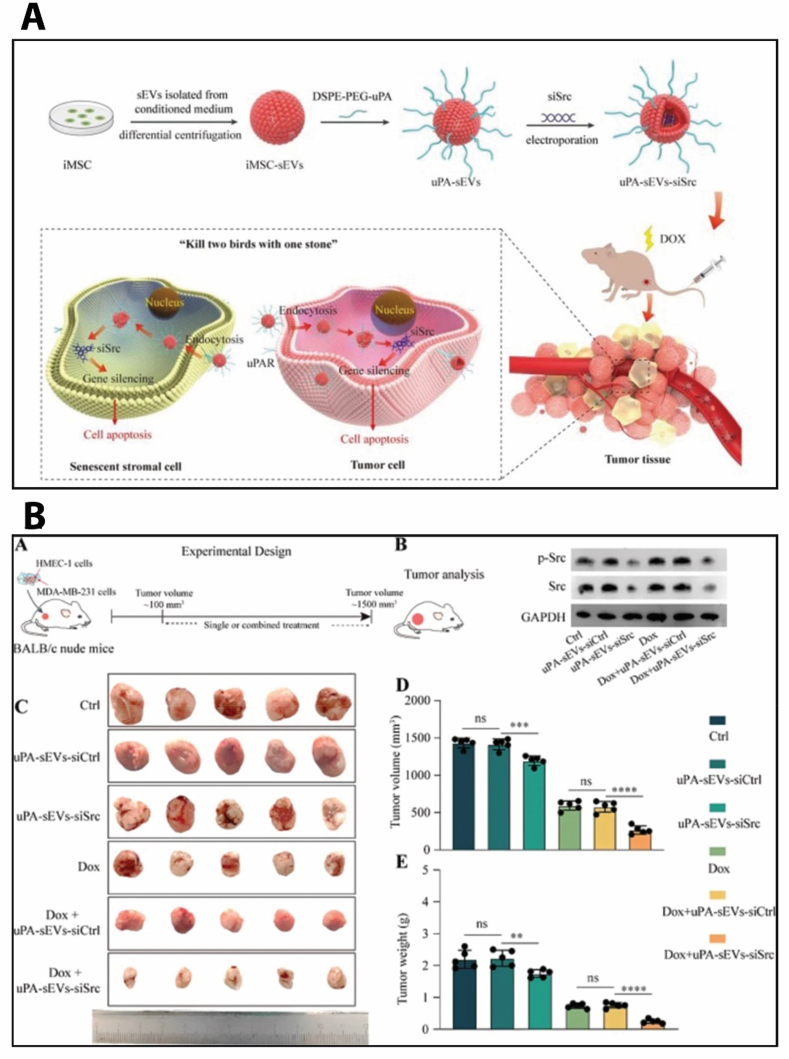
UPA-sEV-siSrc preparation and therapeutic effects on *in vivo* antitumor activity. (A) An outline of uPA-sEVs-siSrc development showing the generation, modification, and functionalization. (B) The schematic illustrates the *in vivo* study designed to evaluate uPA-sEVs-siSrc's antitumor effects. Protein analysis indicated reduced *p*-Src and Src levels, which correlate with marked tumor growth inhibition after uPA-sEVs-siSrc treatment. Tumor images, along with volume and weight measurements, confirmed the superior antitumor efficacy of uPA-sEVs-siSrc compared to other treatments^250^. Reprinted with permission from Ref. 250. Copyright © 2024, Springer Nature.

EVs are excellent nanocarriers that can deliver both therapeutic nucleic acids and small-molecule drugs simultaneously, boosting treatment effectiveness and reducing side effects through improved targeting^213^. Altanerova et al.^214^ engineered cancer cell exosomes with suicide genes (yCD:UPRT) to convert the prodrug 5-fluorocytosine (5-FC) into the toxic drug 5-FU inside cancer cells. MSC-derived exosomes have tumor-homing properties that effectively trigger cancer cell death by converting 5-FC to 5-FU and 5-FUMP, while normal cells remain unaffected. This highlights the potential of exosomes from cells transduced with suicide genes for highly selective drug delivery targeting cancer cells in therapeutic applications. Additionally, the engineering of folate-functionalized microvesicles promotes dual delivery of Bcl-2 siRNA and paclitaxel to cancer cells with high levels of folate receptors, enhancing antitumor effects through Bcl-2 downregulation and the cytotoxicity of paclitaxel. Chiang et al.^215^ also designed a therapeutic EV for treating pancreatic ductal adenocarcinoma (PDAC), which carried a high dose of KRAS^G12D^-silenced RNA or *TP5*3 mRNA and a low dose of gemcitabine (GEM). Furthermore, engineered EVs selectively target senescent stromal cells and neoplastic cells, leading to reduced tumor growth. This method involves integrating Src-siRNA (siSrc) into EVs and enhancing their targeting efficiency by modifying them with uPA peptides. The uPA-sEVs-siSrc system triggered apoptosis in aged stromal cells and neoplastic cells by decreasing senescence load in the tumor xenograft model with reduced tumor progression^216^^,^^217^. These findings highlight the ability of EVs to improve the effectiveness of combination chemotherapy and gene therapy (Figure 14, Figure 15).

### 8.3 Modified EVs for the combination of immunotherapy with chemotherapy

Cancer immunotherapy has transformed oncology by increasing the ability of the immune system to recognize and eliminate cancer cells^218^. Immune cells, such as macrophages, neutrophils, Treg cells, and Th17 cells, can both promote and suppress tumor growth, reflecting their dual roles in tumor progression^219^. For example, M1 macrophages suppress tumor growth while M2 macrophages may reduce antitumor activity and promote tumor progression depending on the TME^220^^,^^221^. Studies demonstrate that chemotherapy can alter tumor antigens to mark cancer cells as threats, causing temporary lymphodepletion, further removing immunosuppressive cells like Tregs and MDSCs^222^, ^223^, ^224^, ^225^, ^226^, ^227^, ^228^, ^229^, ^230^, ^231^, ^232^, ^233^, ^234^, ^235^, ^236^, ^237^, ^238^, ^239^, ^240^, ^241^. Considering these benefits, combining immunotherapy with chemotherapy to amplify synergistic antitumor effects is a promising strategy^223^.

**Figure 16:**
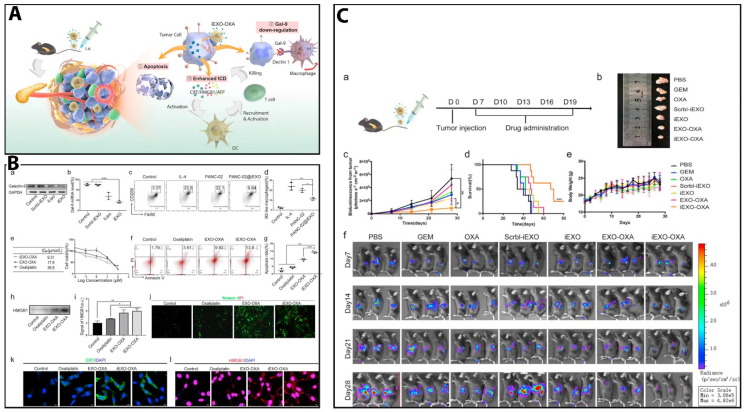
*In vitro* and *in vivo* assessment of iEXO-OXA formulations in anticancer therapy. (A) A schematic illustration of the overall studies. (B) *In vitro* studies showed that iEXO-OXA formulations reduced galectin-9 expression in PANC-02 cells and influenced macrophage polarization, indicating immunomodulatory effects on the tumor microenvironment. These formulations inhibited cancer cell proliferation, lowered IC_50_ values, and promoted apoptosis, as confirmed by flow cytometry, fluorescence microscopy, and confocal laser scanning microscopy (CLSM). They also triggered cellular stress responses, including CRT exposure and HMGB1 secretion, further supporting their anticancer potential. (C) The *in vivo* studies schedule detailed the administration of various formulations over four weeks. iEXO-OXA treatments significantly suppressed tumor growth, as shown by tumor size measurements, bioluminescence imaging, and statistical data, while also improving survival rates. Body weight monitoring indicated good tolerability, and IVIS imaging over the treatment period confirmed their sustained antitumor effects^264^. Reprinted with permission from Ref. 264. Copyright © 2020, Elsevier.

Additionally, EVs can serve as codelivery platforms for immunomodulatory and chemotherapeutic materials, enhancing tumor area accumulation and endocytosis/phagocytosis to reprogram immune responses^224^^,^^225^. For example, Taieb et al.^226^ reported that dendritic cell-derived exosomes improved the antitumor effects of cyclophosphamide in a cancer model. This enhances the anticancer secondary impact of exosomes and eliminates the Treg-mediated suppression of CD4^+^CD25^+^Foxp3^+^ T cells. Prior studies have focused on improving chemo-immunotherapy for metachronous peritoneal carcinomatosis by combining hyperthermic intraperitoneal chemotherapy (HIPEC) with standard treatment. This approach utilizes hybrid nanoparticles that consist of exosomes and thermosensitive liposomes to deliver granulocyte‒macrophage colony-stimulating factor (GM-CSF) and docetaxel, resulting in improved drug penetration and cargo release under hyperthermic conditions induced by HIPEC^227^. Engineered bone marrow-derived mesenchymal stem cells (BM-MSC) exosomes co-delivered an oxaliplatin prodrug and galectin-9 siRNA, improved cancer targeting, altered macrophages to an antitumoral phenotype with reduced Treg function and galectin-9 levels, and increased cytotoxic T lymphocytes (CTLs)^228^. These studies emphasize the application of modified EVs in combination with chemotherapy and immunotherapy for advanced cancer treatment (Fig. 16)^229^.

### 8.4 Modified EVs for the combined delivery of nanoparticles and chemotherapy

**Figure 17:**
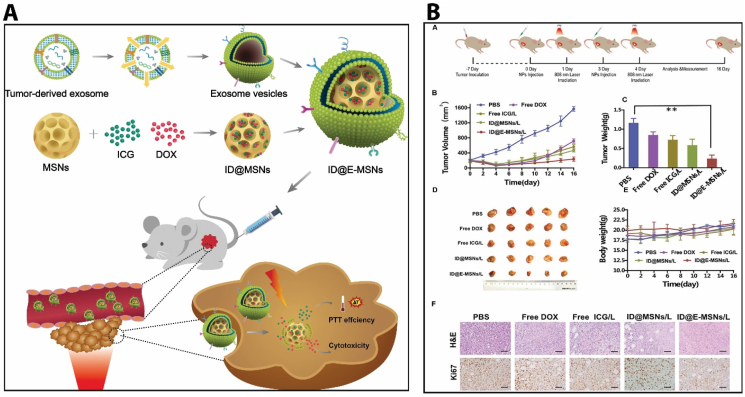
Schematic illustration of tumor-derived exosome-camouflaged nanovehicles for chemo-hotothermal therapy and their combined therapeutic effects *in vivo*. (A) This illustrates a novel cancer therapy combining tumor cell-derived exosome-coated porous silicon nanoparticles with DOX for targeted chemo-photothermal treatment. The combination of chemotherapy and photothermal therapy has improved treatment precision and effectiveness. (B) A schematic illustrates a 16-day treatment with ID@E-MSNs, showing tumor volume curves that highlight combination therapy as an effective treatment. Photographs and tumor weight measurements from different groups visually and quantitatively confirm these results. Body weight trends indicate good treatment tolerability. Histological analysis with HE and Ki67 staining reveals reduced tumor proliferation and tissue changes after treatment. (*n* = 5), (scale bar = 1 mm)^278^. Reprinted with permission from Ref. 278. Copyright © 2020, Frontiers in Bioengineering and Biotechnology.

**Figure 18:**
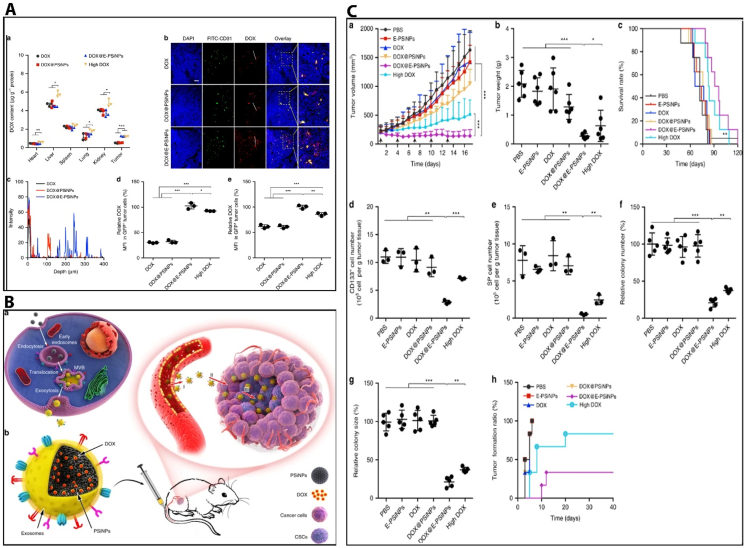
Analysis of DOX distribution, accumulation, and anticancer activity in Tumor Tissue. (A) DOX fluorescence intensity was measured in GFP-positive tumor cells and side population cells, showing varied drug uptake and accumulation across different tumor cell types depending on the DOX formulation and dosage used. (B) The study assessed DOX accumulation in organs and tumors of H22 tumor-bearing mice after intravenous delivery of different DOX formulations and doses, offering insights into the drug biodistribution. (C) Tumor weights at the end of the growth inhibition study showed that DOX@E-PSiNPs had superior anticancer effects compared to other treatments (mean ± SD, *n* = 6). A Kaplan–Meier survival analysis of 4T1 tumor-bearing mice (*n* = 8) demonstrated improved survival with DOX@E-PSiNPs. Additionally, tumor cells from treated mice formed fewer and smaller colonies in 3D fibrin gels, further confirming the formulation's ability to inhibit tumor growth (mean ± SD, *n* = 6)^234^. Reprinted with permission from Ref. 234. Copyright © 2019, Nature Communications.

NPs have a high surface area-to-volume ratio and small size, which endows them with significant loading capacity and makes them excellent tools for tumor treatment^230^^,^^231^. Their nanoscale size enables them to accumulate at the tumor site, further enhancing the permeability and retention effect. Their small size also protects drugs from degradation and aggregation in an acidic TME^230^^,^^231^. NPs can improve drug efficacy by increasing drug accumulation in tumor cells and facilitating the targeted release of drugs in response to specific stimuli, such as changes in pH or temperature^232^, ^233^, ^234^. This approach increased the effectiveness of chemotherapy and minimized side effects to tumor cells. EVs can be engineered to deliver therapeutic NPs, such as gold and magnetic nanoparticles, in combination with chemotherapeutic agents^106^^,^^135^^,^^235^. Despite advancements in cancer therapy for delivering chemotherapy drugs, the use of NPs is constrained by major challenges, including toxicity, immunogenicity, and inadequate targeting ability, which limit their effectiveness and clinical implementation^236^^,^^237^. According to various studies, engineered EVs combined with NPs can alter and improve the physicochemical properties of NPs, increasing their effectiveness in targeting tumor cells^238^^,^^239^. Compared with unmodified NPs, NPs modified with cell membranes or designed as biomimetic NPs tend to have reduced toxicity, lower immunogenicity, longer circulation times, and improved targeting capabilities^238^^,^^240^^,^^241^. For example, a study developed porous silicon NPs coated with exosomes to deliver ICG and Dox. The exosome membrane enhanced the targeting specificity of these biomimetic NPs for cancer cells, resulting in greater accumulation in tumor tissue (Fig. 17)^242^. Studies have shown that exosome-sheathed DOX-loaded porous silicon nanoparticles (Dox@E-PSiNPs) have high tumor accumulation and deep penetration into the tumor parenchyma, thereby enhancing cellular uptake for more effective cancer chemotherapy (Fig. 18)^200^. Studies have also shown that DOX-loaded NPs coated with exosomes derived from endothelial cells can utilize immunogenicity to treat glioblastoma. The results demonstrated that cells effectively take up DOX, which increases brain penetration by crossing the blood‒brain barrier more efficiently^239^. Additionally, surface-functionalized exosomes with a peptide improve the targeting of mesenchymal‒epithelial transition factor (c-Met) in triple-negative breast cancer. Engineered macrophage exosome-coated NPs significantly improve the antitumor efficacy of DOX by increasing the targeting and cellular uptake efficiency^240^. In conclusion, the combination of NPs with exosomes is a feasible approach to increase the efficiency of chemotherapy with minimized side effects.

## 9 Engineered EVs for immunotherapy-related cancer combination therapy

### 9.1 Theories of EVs for immunotherapy-related cancer combination therapy

EVs engineered for immunotherapy-related cancer combination therapy employ various strategies, such as nanovaccine development, immune reprogramming, immune checkpoint blockade, and cytotoxic actions^243^, ^244^, ^245^, ^246^. However, there is no clear mechanism for these approaches^140^^,^^192^^,^^247^. Nanovaccination approach utilizes cancer-related antigens carried by EVs for triggering an adaptive anti-cancer immunity^140^^,^^192^^,^^248^. For example, dendritic cell-derived EVs can display tumor antigens to T cells, initiating an antitumor immune response^249^, ^250^, ^251^. Additionally, the immune-reprogramming strategy involves the use of EVs to deliver immune modulators, such as cytokines or immune checkpoint inhibitors, to reprogram the TME and counteract immunosuppression^140^^,^^192^^,^^252^. For example, interleukin-12-loaded EVs activate NK cells and macrophages, amplifying tumor-cell killing^249^^,^^253^. Moreover, EV-mediated checkpoint blockade interrupts the immune checkpoint molecules/ligands interactions, which are typically upregulated in cancer cells to enable immune escape^140^^,^^192^^,^^254^^,^^255^. For example, EVs loaded with anti-PD-L1 antibodies can inhibit the PD-1/PD-L1 pathway, restoring T-cell cytotoxity^249^^,^^256^.

**Figure 19:**
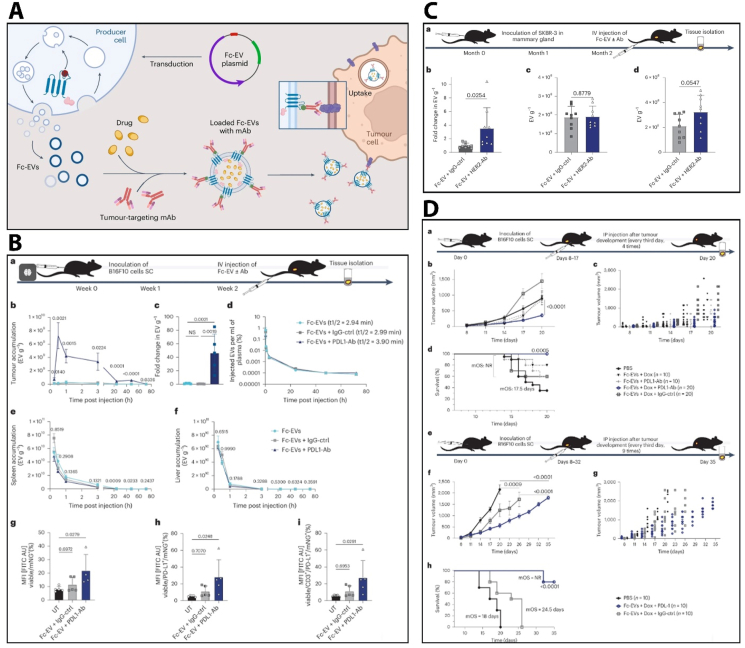
The therapeutic potential of intravenously injected EVs in cancer treatment. (A) Development of EVs by using the Fc-binding domain (Fc-EVs) designed to deliver antibodies and therapeutic agents directly to cancer cells. (B) Quantified EVs in tumor tissue, plasma, spleen, and liver over time postinjection. The flow cytometry analysis of the uptake of Fc-EVs labeled with mNeonGreen (mNG) and PD-L1 antibody (PD-L1-Ab) or control antibody (Control-Ab) by tumor cells, indicating the percentages of tumor cells and immune cells taken up by the EVs. (C) The experimental setup involved EV injections for breast cancer treatment. Results showed changes in EV levels and their accumulation in the spleen and liver. (D) The treatment schedule, tumor volume measurements, individual tumor growth, and survival rates across different groups demonstrated significant tumor suppression and improved survival with certain EV therapies. Data are shown as mean ± SD for 5–20 mice, with statistical significance noted^10^. Reprinted with permission from Ref. 10. Copyright © 2024, Nature Biomedical Engineering.

Cytotoxic EV strategies deliver chemotherapeutics, gene regulators, or nanomaterials to kill or sensitize cancer cells^116^^,^^140^^,^^192^. For example, doxorubicin-loaded EVs induce apoptosis in breast cancer cells^200^^,^^249^. This approach harnesses EV properties to target specific cells with therapeutic agents, providing a safer and more personalized modality. Studies have utilized different delivery systems to target cancer cells with HER2 or PD-L1 receptors, demonstrating their effectiveness in a melanoma mouse model (Fig. 19)^6^.

Beyond drug delivery, engineered EVs can increase the efficacy of adoptive cell therapy, such as CAR-T cell therapy^257^, ^258^, ^259^, ^260^. EVs bearing tumor-specific antigens or costimulatory molecules improve CAR-T cell targeting and activation^257^^,^^261^. Moreover, combining immunotherapy with cytotoxic agents or photothermal strategies may further enhance treatment outcomes^141^. For example, Li et al.^141^^,^^262^ developed HSP70-enriched extracellular vesicles loaded with tellurium nanoparticles for light-driven tumor immunotherapy. The engineered vesicles promoted antigen presentation and exerted photothermal activity, thereby amplifying anticancer immune responses. As a result, this platform improved the efficacy of tumor immunotherapy under light irradiation. These findings further emphasize the potential of engineered EV-based nanoplatforms in advanced cancer therapy.

### 9.2 Mechanisms of modified EVs for combination therapy related to immunotherapy

#### 9.2.1 Cell-to-cell communication

**Figure 20:**
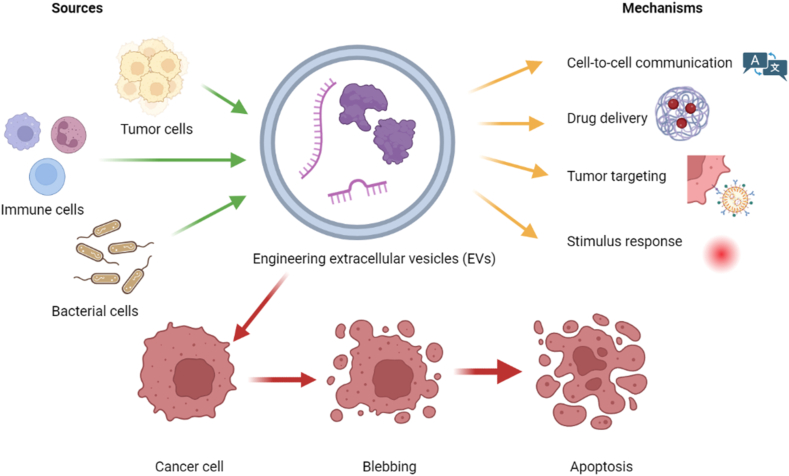
EVs engineered for cancer immunotherapy and combination treatments can be created using tumor cells, immune cells, or bacteria. These engineered EVs are designed to facilitate cell-to-cell communication, deliver drugs, target tumors, and respond to specific stimuli, enhancing their therapeutic potential.

Engineered EVs facilitate protein, lipid, and nucleic acid transfer, influencing signaling pathways and gene expression in recipient cells^20^^,^^192^^,^^249^^,^^263^. EVs from immune cells can modulate the immune response or regulate cancer cell characteristics (Fig. 20)^264^.

#### 9.2.2 Drug delivery

Engineered EVs efficiently deliver agents such as chemotherapeutic drugs, gene regulators, or immune modulators to target cells (Fig. 20)^140^^,^^191^^,^^192^^,^^249^^,^^265^. For example, EVs carrying DOX can induce apoptosis in breast cancer cells^198^.

#### 9.2.3 Tumor targeting

Engineered EVs modified with specific ligands or antibodies can target cancer or tumor-associated cells (Fig. 20)^192^^,^^228^^,^^249^. For example, EVs modified with anti-HER2 antibodies can target HER2-positive breast cancer cells^192^^,^^240^.

#### 9.2.4 Stimulus response

Engineered EVs can be designed to respond to stimuli such as pH, temperature, light, or enzymes, enabling controlled release of their cargo (Fig. 20)^249^^,^^266^^,^^267^.

### 9.3 Sources of EVs for immunotherapy-related cancer combination therapy

EVs are naturally released by a vast array of cell types across different domains of life, including prokaryotic and eukaryotic cells (Table 5)^268^. Their presence is widespread in various biological fluids, making them readily accessible for study and potential therapeutic use^268^, ^269^, ^270^.

**Table 5:** Comparative characteristics of EVs from different sources for cancer therapy.

Source	Typical constituent	Advantage	Limitation
Cancer Cells	PD-L1, TGF-*β*, miRNAs, sphingomyelin, cholesterol	High tumor tropism	Oncogenic cargo; immunosuppression
Immune Cells	MHC I/II, cytokines, miRNAs	Activate antitumor immunity	Limited yield; source variability
MSCs	Tetraspanins, growth factors, miRNAs	Low immunogenicity; TME modulation	Possible tumor promotion; scalability issues
Body fluids	ctDNA, miRNAs, proteins, lipids	Minimally invasive; accessible	Heterogeneous content; Contamination risk
Milk	Proteins, si/miRNA, phospholipids	Oral delivery; biocompatible	Species variability; Cargo stability
Plants	Bioactive compounds, miRNAs, lipids	Scalable; oral tolerance	Cross-kingdom transfer; stability issues
Bacteria	Immune-stimulating proteins, nucleic acids	Strong immune stimulation; Customizable	Safety concerns; purification challenges

TME, tumor microenvironment; ctDNA, circulating tumor DNA; MHC, major histocompatibility complex.

#### 9.3.1 Mammalian cell sources

A primary source of EVs is mammalian cells, which include immune cells (like lymphocytes and dendritic cells), stem cells, cancer cells (which can produce “oncosomes”), somatic cells (such as epithelial cells, endothelial cells, and neurons), platelets, and red blood cells, which are known to secrete EVs^268^, ^269^, ^270^, ^271^. These EVs can be recoverable from conditioned medium supernatants or directly from biofluids^269^^,^^271^. Human body fluids such as blood (plasma/serum), saliva, urine, cerebrospinal fluid, amniotic fluid, breast milk, synovial fluid, tears, lymph, bile, and gastric acid are rich reservoirs of EVs^268^, ^269^, ^270^^,^^272^. Interestingly, studies on human plasma suggest that a vast majority (around 99.8%) of circulating EVs originate from hematopoietic cells, with a smaller fraction derived from other tissues^268^. Body fluids offer a natural source, though they have limited availability for large-scale EV production. Hence, commercialized cell lines are often considered a more scalable option^269^. Platelet-derived exosomes (PL-EXOs) are useful in regenerative medicine, wound healing, and disease progression by delivering growth factors, miRNAs, and signaling molecules to target cells^273^. They also contribute to atherosclerosis, cancer, and liver fibrosis through intercellular communication while also serving as potential biomarkers in retinal diseases^273^. Easily isolated from platelet-rich plasma (PRP) and stable at −80 °C, PL-EXOs offer therapeutic advantages with low immunogenicity and tumorigenesis risks^274^. Engineered PL-EXOs enhance drug delivery by evading immune clearance, targeting tumors *via* P-selectin, and releasing payloads in acidic microenvironments, demonstrating promise for precision medicine and anti-tumor therapies^273^.

#### 9.3.2 Non-mammalian and pathogen sources

Beyond mammalian cells, EVs are also produced by a diverse range of non-mammalian organisms. These include plants, bacteria (both gram-negative and gram-positive), fungi, and parasites^268^^,^^269^^,^^271^^,^^275^. Plant-derived nanovesicles and milk-derived exosomes (*e.g.*, from bovine milk) are being explored for their therapeutic potential, particularly in cancer treatment, and are considered suitable for large-scale production^269^. Bacterial EVs, which can arise from the outward budding of the outer membrane in gram-negative bacteria or the cytoplasmic membrane in gram-positive bacteria, are involved in processes like horizontal gene transfer and modulation of host immunity^268^. The secretion of EVs by pathogenic entities like bacteria and viruses is a field of growing interest, as these vesicles can carry virulence factors and play roles in pathogenesis^268^. The universal nature of EV secretion is highlighted by their production even by archaeal cells.

### 9.4 Methodologies for EV isolation, characterization, and engineering

The successful therapeutic application of EVs depends on precise and reproducible methods for their isolation, characterization, and engineering^276^^,^^277^. Several isolation techniques are currently employed, each offering distinct advantages and limitations^278^. Differential ultracentrifugation remains the gold standard, relying on sequential high-speed centrifugation steps to pellet EVs from conditioned media or biological fluids^279^. Although widely adopted, this technique is time-consuming, yields low throughput, and may co-isolate protein aggregates and other contaminants^280^. Size-exclusion chromatography (SEC), which separates particles based on size as they pass through a porous matrix, has emerged as a gentler and more reproducible alternative, offering higher purity for clinical-grade applications^281^.

Tangential flow filtration (TFF) is gaining traction as a scalable method suitable for large-volume EV production under good manufacturing practice (GMP) conditions^282^^,^^283^. This technique concentrates EVs while removing smaller impurities using semi-permeable membranes^279^. Meanwhile, precipitation-based commercial kits, such as ExoQuick, offer user-friendly workflows but often result in lower purity due to the co-precipitation of non-vesicular materials^281^. More recently, microfluidic-based isolation platforms have been developed for high-precision EV separation using size, charge, or affinity-based mechanisms^284^.

Characterization of EVs is essential to confirm their identity, ensure batch consistency, and assess therapeutic potential^285^. Nanoparticle tracking analysis (NTA) is widely used to measure EV size distribution and concentration by tracking the Brownian motion of individual vesicles^286^. Transmission electron microscopy (TEM) provides high-resolution visualization of EV morphology, complementing NTA in confirming vesicle identity^287^^,^^288^. Western blotting and flow cytometry are routinely employed to detect EV-specific markers such as CD9, CD63, CD81, ALIX, and TSG101^289^. In some applications, bead-based flow cytometry enables the detection of surface markers on smaller EVs^290^. Additional characterization techniques include dynamic light scattering (DLS) for size and zeta potential analysis, and molecular profiling methods such as quantitative PCR, RNA sequencing, or mass spectrometry to evaluate RNA and protein cargo composition^291^^,^^292^.

EVs can be engineered through various functionalization strategies to enhance their therapeutic efficacy and specificity^293^. For cargo loading, passive methods such as simple incubation are used for small molecules and hydrophobic compounds, while active methods like electroporation, sonication, and extrusion can facilitate the encapsulation of nucleic acids, proteins, or gene-editing complexes into EVs^294^^,^^295^. Surface engineering involves the chemical modification of EV membranes using click chemistry, lipid insertion, or ligand conjugation to improve targeting specificity or enable imaging^98^^,^^296^^,^^297^. Additionally, parental cell engineering where donor cells are genetically modified to overexpress desired therapeutic molecules or targeting moieties, offers a powerful strategy to control EV content from the source^298^^,^^299^. To ensure clinical relevance, EV production workflows must adhere to rigorous quality control measures^300^^,^^301^. The Minimum Information for Studies of Extracellular Vesicles (MISEV) 2018 and 2023 guidelines recommend standardized criteria for EV identification, including both physical properties and molecular markers^302^^,^^303^. Batch-to-batch assessment of purity, potency, and functional activity is essential for reproducibility and regulatory approval^304^. As EV therapies progress toward the clinic, harmonizing isolation, characterization, and engineering protocols remains a top priority to facilitate large-scale manufacturing and ensure patient safety^301^.

## 10 Perspectives and future directions

Engineered EVs for pharmacological delivery have emerged as a promising strategy for oncology and other diseases. Over the past decade, combining anticancer drugs with other therapies has advanced cancer treatment significantly. Compared to single treatments, combination therapy more effectively targets multiple pathways, helping to reduce drug resistance, slow tumor growth, and prevent metastasis^214^^,^^305^. Moreover, advancements in EVs and their engineered forms have been characterized by high loading capacity, biocompatibility, and precise tumor-targeting abilities, establishing them as promising platforms for combination therapy in cancer^306^. Despite the high therapeutic potential of engineered EVs, there are critical challenges in their characterization and mass production that must be addressed to make them viable as off-the-shelf products^307^. The development of scalable and cost–effective methods while preserving the composition and potency of EVs is crucial for EV-based therapeutics. Significant obstacles in clinical trials include low EV production yields, the lack of large-scale and clinical-grade purification protocols, and the heterogeneity of EVs due to their varied sizes and contents^307^^,^^308^. Additionally, modifications to reduce immune clearance, improve targeting, and enhance tropism for site-specific drug delivery are essential for the therapeutic potential of Ev^309^.

In clinical trials, various sources of EVs have been found to offer distinct therapeutic potential^310^^,^^311^. For example, blood- and milk-derived EVs are used for drug delivery^312^^,^^313^. EVs obtained from dendritic cells, mesenchymal stem cells, and patient-derived tumor cells have been used in clinical trials^314^, ^315^, ^316^. However, the production and application of EVs derived from immune cells pose significant challenges, which limit their therapeutic use despite their potential^317^. Novel technologies and methods have been developed to facilitate scalable biomanufacturing of EVs^318^. Strategies such as modifying the culture environment to stimulate mass EV production, inducing hypoxia, increasing intracellular calcium levels, inducing serum starvation, and applying mechanical stress are employed^319^^,^^320^. Hollow fiber and other various bioreactors have been designed for continuous cell culture to facilitate large-scale EV production^320^^,^^321^. For precise and accurate analysis of EV morphology and cargo, advanced techniques such as atomic force microscopy (AFM), small-angle X-ray scattering (SAXS), and super-resolution fluorescence microscopy have been developed^322^, ^323^, ^324^. Furthermore, specific loading strategies and modification techniques either applied before or after isolation can be used to direct EVs to target cells^325^.

EVs are increasingly recognized for their potential in sophisticated therapeutic applications, particularly through multiplex engineering, which aims to equip them with multiple functionalities like targeted drug delivery, the ability to perform sequential therapeutic actions, and features for real-time imaging^6^^,^^326^. These naturally occurring, cell-derived nanoparticles are adept at intercellular communication and can transport bioactive molecules, making them suitable for complex medical interventions^327^, ^328^, ^329^. The core idea behind EV multiplexing is to modify these vesicles to carry diverse therapeutic cargoes, direct them to specific cells or tissues, and integrate imaging agents for monitoring their activity and distribution^6^^,^^326^.

Multiplex engineering encompasses several strategies, primarily focusing on cargo loading and surface modification. EVs can be loaded with a variety of therapeutic agents, including small-molecule drugs, proteins, and nucleic acids, using either endogenous methods (genetically modifying producer cells) or exogenous techniques (direct loading into isolated EVs)^326^. This allows for the co-delivery of multiple drugs or the combination of therapeutics with imaging probes within a single EV^326^. Surface engineering involves adorning the EV surface with specific ligands, such as antibodies or peptides, to recognize and bind to target cells, thereby enhancing therapeutic precision and minimizing off-target effects^6^^,^^326^. For example, EVs can be engineered to display antibodies targeting cancer cell markers or Fc-binding domains that allow for flexible attachment of various antibodies, enabling targeted delivery to diverse tissues^6^. The concept of a single EV releasing multiple drugs in a precisely programmed sequence is advanced. Hence, engineered EVs can facilitate these sequential therapeutic effects. This can be achieved through strategies like delivering engineered transcription factors *via* EVs to modulate gene expression in target cells, leading to a subsequent, programmed cellular response^326^. Another approach involves the coordinated use of different EV populations, each engineered with distinct cargoes and release profiles, administered in a timed sequence. Furthermore, EVs can be co-loaded with a therapeutic agent and an imaging agent, allowing the drug's action to be monitored concurrently with its delivery and accumulation at the target site, effectively enabling imaging-guided therapy^326^.

The ability to track and visualize engineered EVs *in vivo* is critical for evaluating their biodistribution, target engagement, and therapeutic efficacy^326^. Imaging techniques include direct labeling of EVs with fluorescent dyes, radionuclides, or magnetic particles, and indirect labeling, where producer cells are engineered to express reporter genes (like luciferase) that are then incorporated into the released EVs^326^. These methods, utilizing imaging modalities such as fluorescence, bioluminescence, and nuclear imaging (SPECT/PET), provide vital feedback for optimizing EV-based therapies and confirming their action in preclinical settings. The choice of imaging strategy is often guided by the required sensitivity, resolution, and depth of penetration for the specific application^326^. Technologies for multiplexed profiling of EVs, including at the single-vesicle level, are also advancing, offering deeper insights into EV heterogeneity and function, which is crucial for developing effective EV-based biomarkers and therapeutics^326^^,^^328^^,^^330^^,^^331^.

The integration of Artificial Intelligence (AI) with EV research is paving the way for more precise and personalized EV-based therapies^332^^,^^333^. AI can analyze vast and complex biological datasets, offering significant advantages in overcoming challenges associated with EV heterogeneity and in optimizing their design for specific therapeutic applications^332^. This predictive tool is crucial for the rational design of engineered EVs. For instance, AI can help in selecting optimal donor cells, culture conditions, and engineering strategies (*e.g.*, surface modifications or cargo loading techniques) to produce EVs with desired therapeutic properties tailored to an individual patient's disease profile^332^. By integrating patient-specific genetics, EV biomarkers, and clinical data, AI guides personalized EV treatment plans^332^^,^^334^. AI enhances the accuracy of EV-based diagnostics, aiding the development of personalized therapy. For instance, machine learning paired with EV microscale cytometry (*e.g.*, the EVMAP platform) can identify disease-specific EV biomarkers in patient samples to build predictive models for cancer^335^. For example, AI can assist in analyzing EV proteomics data to identify rare proteins that could serve as targets or therapeutic payloads^336^. AI also plays a role in optimizing drug delivery systems. It can be used to predict how engineered EVs will interact with target cells and tissues, their biodistribution, and their efficacy in delivering therapeutic cargo^221^^,^^332^. This includes guiding the design of EVs for controlled drug release and real-time monitoring, potentially leading to AI-directed EV therapies where treatment parameters are dynamically adjusted based on real-time feedback^221^^,^^337^. The overarching goal is to leverage AI to make EV-based therapies more targeted, efficient, and tailored to the unique biological landscape of each patient, thereby advancing the field of personalized medicine^333^^,^^337^. However, standardization of protocols for EV production, isolation, and characterization, as well as addressing the challenge of EV heterogeneity, remains a critical consideration for the successful clinical translation of AI-guided EV therapies.

A primary challenge in using EVs is the inherent heterogeneity of EVs and the difficulties in standardizing their production and characterization^338^^,^^339^. EVs can vary significantly based on the parent cell type and even culture conditions, leading to batch-to-batch differences in composition, stability, and therapeutic activity^8^^,^^333^. This variability makes it challenging to establish consistent manufacturing processes, ensure product quality, and scale up production for clinical demands^302^^,^^339^^,^^340^. Specifically, achieving efficient and consistent loading of therapeutic cargo into engineered EVs and purifying large quantities of homogeneous vesicles remain critical manufacturing obstacles^339^^,^^340^. Technologies like lyophilization and cryopreservation are being explored to improve stability, but further investigation is needed to confirm that these processes do not alter EV properties^338^. Additionally, the exact mechanisms behind many EV-based therapies remain unclear, making it challenging to establish proper quality control and potency tests needed for regulatory approval^338^. Furthermore, key pharmacological factors for EVs, such as optimal dosing, administration routes, biodistribution, and pharmacokinetics, are often poorly understood. Challenges such as low drug load per EV, inefficient uptake by target cells, cargo degradation, and rapid clearance by the immune system can reduce effectiveness. Additionally, long-term safety and immunogenicity require thorough evaluation^302^. Meanwhile, the variability and potential impurity of EV preparations are of significant concern^216^.

The regulatory landscape for EV-based therapeutics is complex and still evolving, varying across different countries^216^. In the United States, the Food and Drug Administration (FDA) regulates EV products as drugs and/or biological products, requiring rigorous premarket review and approval^216^^,^^341^. As of late 2023 and early 2024, no EV products had received FDA approval for therapeutic use, and the FDA has issued warnings regarding unapproved EV products being marketed to consumers^216^. The lack of comprehensive regulatory guidance specifically tailored to EV-based products further complicates the approval process^153^. Establishing industry standards for manufacturing, quality control (including potency testing), and the professional knowledge required for handling these cellular products are necessary steps for ensuring quality and safety^216^^,^^333^. While there have been some recent approvals of cell-based therapies like Ryoncil (remestemcel-L) for certain conditions, which may offer insights for the EV field, significant challenges related to batch variability and potency testing persist for both cell and EV products^333^.

## 11 Conclusions

This review highlights the synergistic potential of engineered EVs combined with various cancer therapies and emphasizes the increasing preclinical evidence supporting this integrated approach as a promising avenue in oncology. The combination of engineered EVs with conventional or emerging treatments such as chemotherapy, radiotherapy, immunotherapy, and targeted therapies may enhance therapeutic efficacy, reduce side effects, and overcome drug resistance. Despite this promise, most of the current research remains at the preclinical stage. Therefore, robust, scalable, and clinically viable strategies are urgently required to translate engineered EV-cancer therapy combinations into routine oncology practice.

## Author contributions

Anita S. La'ah: Writing – original draft, Writing – review & editing. Nashmin Fayazi Hosseini: Writing – original draft. Choongmo Kang: Writing – original draft. Amirhossein Ahmadieh-Yazdi: Writing – original draft. Mohsen Sheykhhasan: Conceptualization, writing – original draft, writing – review & editing. Piao Yang: Writing – original draft, Writing – review & editing. Armita Ghotaslou: Writing – original draft, Writing – review & editing. Hamed Manoochehri: Writing – original draft, Writing – review & editing, Visualization. Yun Suk Huh: Writing – review & editing. Heemin Kang: Conceptualization, writing – review & editing. Pooyan Makvandi: Conceptualization, writing – original draft, writing – review & editing.

## Conflicts of interest

The authors declare no conflicts of interest.
